# Optimization of cancer immunotherapy through pyroptosis: A pyroptosis-related signature predicts survival benefit and potential synergy for immunotherapy in glioma

**DOI:** 10.3389/fimmu.2022.961933

**Published:** 2022-08-03

**Authors:** Yu Zeng, Yonghua Cai, Peng Chai, Yangqi Mao, Yanwen Chen, Li Wang, Kunlin Zeng, Ziling Zhan, Yuxin Xie, Cuiying Li, Hongchao Zhan, Liqian Zhao, Xiaoxia Chen, Xiaoxia Zhu, Yu Liu, Ming Chen, Ye Song, Aidong Zhou

**Affiliations:** ^1^ Department of Cell Biology, School of Basic Medical Science, Southern Medical University, Guangzhou, China; ^2^ Department of Neurosurgery, Nanfang Hospital, Southern Medical University, Guangzhou, China; ^3^ Department of Radiation Oncology, Zhujiang Hospital, Southern Medical University, Guangzhou, China; ^4^ Department of Neurosurgery, Shanghai Children’s Hospital, Shanghai Jiao Tong University, Shanghai, China; ^5^ Department of Neurosurgery, Xinhua Hospital, School of Medicine, Shanghai Jiao Tong University, Shanghai, China; ^6^ Department of Neurosurgery, Ganzhou People’s Hospital, Ganzhou, China; ^7^ Guangdong Province Key Laboratory of Molecular Tumor Pathology, Southern Medical University, Guangzhou, China

**Keywords:** glioma, pyroptosis, prognosis, tumor-associated microenvironment, immunotherapy, small molecular inhibitor

## Abstract

**Background:**

Pyroptosis is a critical type of programmed cell death that is strongly associated with the regulation of tumor and immune cell functions. However, the role of pyroptosis in tumor progression and remodeling of the tumor microenvironment in gliomas has not been extensively studied. Thus, in this study, we aimed to establish a comprehensive pyroptosis-related signature and uncover its potential clinical application in gliomas.

**Methods:**

The TCGA glioma cohort was obtained and divided into training and internal validation cohorts, while the CGGA glioma cohort was used as an external validation cohort. Unsupervised consensus clustering was performed to identify pyroptosis-related expression patterns. A Cox regression analysis was performed to establish a pyroptosis-related risk signature. Real-time quantitative PCR was performed to analyze the expression of signature genes in glioma tissues. Immune infiltration was analyzed and validated by immunohistochemical staining. The expression patterns of signature genes in different cell types were analyzed using single-cell RNA sequencing data. Finally, therapeutic responses to chemotherapy, immunotherapy, and potential small-molecule inhibitors were investigated.

**Results:**

Patients with glioma were stratified into clusters 1 and 2 based on the expression patterns of pyroptosis-related genes. Cluster 2 showed a longer overall (P<0.001) and progression-free survival time (P<0.001) than Cluster 1. CD8+ T cell enrichment was observed in Cluster 1. A pyroptosis-related risk signature (PRRS) was then established. The high PRRS group showed a significantly poorer prognosis than the low PRRS group in the training cohort (P<0.001), with validation in the internal and external validation cohorts. Immunohistochemical staining demonstrated that CD8+ T cells were enriched in high PRRS glioma tissues. PRRS genes also showed cell-specific expression in tumor and immune cells. Moreover, the high PRRS risk group showed higher temozolomide sensitivity and increased response to anti-PD1 treatment in a glioblastoma immunotherapy cohort. Finally, Bcl-2 inhibitors were screened as candidates for adjunct immunotherapy of gliomas.

**Conclusion:**

The pyroptosis-related signature established in this study can be used to reliably predict clinical outcomes and immunotherapy responses in glioma patients. The correlation between the pyroptosis signature and the tumor immune microenvironment may be used to further guide the sensitization of glioma patients to immunotherapy.

## Introduction

Pyroptosis is a specific type of programmed cell death characterized by immune activation. It was previously considered a form of apoptosis, since it shares some characteristics with the latter, including caspase-dependence, nuclear condensation with DNA damage, cell swelling, and finally cell death (1). However, D’Souza *et al.* described this pro-inflammatory cell death program in *Salmonella*-infected macrophages, which was distinct from the non-inflammatory cell death observed in apoptosis; they proposed the term *pyroptosis (* 2). Subsequent studies have discovered a canonical mechanism of pyroptosis: activation of interleukin-1 converting enzyme (ICE, also known as Caspase-1), cleaved gasdermin D (GSDMD), pro-IL-1β, and pro-IL-18. The N-terminal domain of GSDMD can oligomerize into the cell membrane to form nonselective pores, leading to cell membrane rupture and release of mature IL-1β and IL-18 (3). Recent studies have further revealed the non-canonical pathways of pyroptosis, involving Caspase-3/4/5/6/8/9/11 and granzymes (4–8). Pyroptosis participates in innate immunity and is associated with infectious and autoimmune diseases, nervous system diseases, and tumor (9). However, controversy remains regarding the role of pyroptosis in cancer, as different types of pyroptosis activation lead to distinct effects in different types of cancer. Therefore, a comprehensive analysis of the different pyroptosis processes in specific types of cancers is needed.

Glioma is the most common primary malignancy of the central nervous system and is characterized by high therapeutic resistance and mortality. This is especially true for glioblastoma (GBM), which is the most malignant type of glioma. Immune checkpoint inhibitors (ICIs), such as anti-CTLA-4 and anti-PD-1/PD-L1 monoclonal antibodies (mAb), have achieved great success in other aggressive malignancies, such as melanoma and non-small cell lung carcinoma (10, 11). Although immunotherapy has achieved considerable success in exceptional cases of recurrent GBM (12, 13), several subsequent clinical trials evaluating anti-PD-1 therapy in newly diagnosed or recurrent GBM have failed to show clinical efficacy (14–16). The low response rate to ICIs observed in GBM may be partly attributed to its immunologically cold state with few T-cell infiltrations and the predominance of immunosuppressive tumor-associated macrophages (TAMs) (16, 17). Hence, inflammatory processes such as pyroptosis may be promising targets to remodel the tumor-associated immune microenvironment in glioma and sensitize patients to immunotherapy.

A comprehensive understanding of the pyroptosis landscape involving both canonical and non-canonical pathways in glioma is still needed. Therefore, in the present study, we aimed to investigate the pyroptosis-related expression pattern, considering both canonical and non-canonical pathways in gliomas, and to validate its value in predicting prognosis and survival benefit from immunotherapy. A cluster model was established with pyroptosis-related genes focused on gene co-expression patterns, and a risk signature was established based on the prognostic subset from the above gene sets and focused specifically on prognostic value and therapy response. Their associations with genomic alterations in tumor driver genes, clinicopathological characteristics, and prognosis were investigated. Immune infiltration patterns in different pyroptosis-related groups were analyzed, and the expression of pyroptosis signature-related genes was confirmed in different cell types using single-cell RNA sequencing data. Furthermore, the relationship between pyroptosis-related signatures and immunotherapy response was predicted and validated in patients with GBM who received immunotherapy. Finally, a potential targeted therapy based on a pyroptosis-related signature that may synergize with immunotherapy was predicted. This comprehensive analysis emphasizes the critical role of pyroptosis in shaping the tumor-associated microenvironment and its potential as a target for optimizing glioma immunotherapy.

## Methods

### Data access and processing

RNA-sequencing data from TCGA-663, CGGA-325, CGGA-693, and CPTAC-GBM cohorts with corresponding clinical information were obtained from The Cancer Genome Atlas (TCGA) database (version 28.0, https://portal.gdc.cancer.gov/), the Chinese Glioma Genome Atlas (CGGA) database (http://www.cgga.org.cn/index.jsp), and the Clinical Proteomic Tumor Analysis Consortium (CPTAC) (18). Transcriptional expression was evaluated using transcripts per million (TPM) and was further normalized to log2 (TPM + 1). The establishment of pyroptosis-related signature and internal validation was performed based on the TCGA-663 cohort, while the CGGA-325, CGGA-693, and CPTAC-GBM cohorts were used for external validation. The baseline clinical characteristics of glioma patients are shown in **Supplementary Table 1**. Mutation and copy number variation data were retrieved from the cBioPortal (http://www.cbioportal.org) for the analysis of driver gene mutations. Mutation data were downloaded from TCGA and visualized using the *maftools* package in R to identify the somatic mutation landscape in distinct pyroptosis-related subtypes.

### Establishment of the pyroptosis-related clusters

To obtain consensus clustering of glioma patients based on genes related to both canonical and non-canonical pyroptosis as previously described (19) (**Supplementary Table 2**), unsupervised clustering was performed using *ConsensusClusterPlus* package in R based on 80% sample resampling for 10 repetitions (20). The optimal number of clusters is determined using an empirical cumulative distribution function plot.

### Establishment of pyroptosis-related risk signature

To establish the pyroptosis-related risk signature (PRRS), univariate and multivariate Cox regression analyses were performed using *survminer* in the R package. Briefly, univariate Cox regression analysis was performed based on 26 pyroptosis-related genes for overall survival. Significant factors (P< 0.05) were then selected for multivariate Cox regression. The risk signature was further calculated based on gene expression and the coefficient in multivariate Cox regression analysis for each sample. Receiver operating characteristic (ROC) curve analysis was performed to assess the prognosis-predicted performance, and the area under the curve (AUC) was calculated using *timeROC* in the R package.

### Functional enrichment analysis based on gene set enrichment Analysis and STRING database

To explore the potential pathways associated with pyroptosis-related signatures, GSEA software (version 3.0) was obtained from the GSEA website (http://software.broadinstitute.org/gsea/index.jsp), and enrichment analysis was then performed based on differentially expressed genes (DEGs) between pyroptosis-related subgroups (Cluster 1 vs. Cluster 2; high-PRRS group vs. low-PRRS group, separated by the median PRRS value), with the minimum number of genes set to 5, the maximum number of genes set to 5000, and 1000 resampling; P values< 0.05, and FDR< 0.25 were considered statistically significant. The results were visualized using *ggplot2* in the R package.

### Analysis of immune characteristics and immune infiltration

To evaluate immune characteristics, the immuneScore and stromalScore were calculated using the *estimate* package in R (21). The tumor mutation burden (TMB) score was calculated using *maftools* package in R (22) and the microsatellite instability (MSI) score was obtained from a previous study to evaluate genomic status (23). To assess the degree of oncogenic differentiation, stemness indices (mRNA expression-based stemness index, mRNAsi) for each sample were calculated using a one-class logistic regression machine learning algorithm as previously described (24). To characterize immune infiltration in glioma tissues, the *immunedeconv* R package, which contains six different algorithms, including TIMER, EPIC, MCP-counter, quanTIseq, CIBERSORT, and xCell, was used (25).

### Collection of glioma samples and real-time quantitative PCR

Twelve clinical glioma samples were obtained from the Department of Neurosurgery, Nanfang Hospital, Southern Medical University, Guangzhou, China. RNA was extracted from glioma tissues using TRIzol reagent and reverse-transcribed (Takara Bio Inc., Shiga, Japan). For RT-qPCR, cDNA was amplified using SYBR Green PCR Master Mix (Yeasen Biotechnology, China), with three independent replicates. Relative mRNA expression levels were normalized to that of β-actin. The primers used for RT-qPCR are listed in **Supplementary Table 3**. Prior consent was obtained from the patients for the use of their clinical materials for research purposes, and approval was obtained from the ethics committees of Nanfang Hospital.

### Immunohistochemistry

Paraffin sections prepared from clinical samples were used for IHC to detect CD8 + T cell infiltration as previously described (26). Mouse anti-CD8-α (cat. No. sc-7970, 1:100; Santa Cruz Biotechnology, CA, USA) and goat anti-mouse secondary antibodies (Cat. No. PV-9000, Zhongshan Jinqiao, Beijing, China) were used. After incubation with the secondary antibody, the sections were visualized with a DAB kit (Cat. No. ZLI-9018, Zhongshan Jinqiao, Beijing, China), counterstained with hematoxylin, and analyzed using a bright-field microscope equipped with a digital camera (Nikon, Japan).

### Analysis of PRRS gene expression pattern with single cell RNA sequencing data

Single-cell RNA (scRNA) sequencing data for glioblastoma were retrieved from a previous study that identified malignant cells as CD45^-^ cells with significant copy number alterations (27). To specifically analyze the PRRS gene expression pattern in tumor-associated macrophages (TAMs), another scRNA dataset focused on CD45^+^ immune cells within glioblastoma tissues was used (28). The data were analyzed using the *seurat* package in R. Briefly, a series of quality filters was applied to the data to remove cells with too few total transcript counts (< 1,000), possible debris with too few genes expressed (< 200), possibly more than one cell with too many genes expressed (> 7,500), too many counts (> 7,500), and possible dead cells or a sign of cellular stress and apoptosis with a high proportion of mitochondrial gene expression over the total transcript counts (> 10%). Clustering was performed on K-nearest neighbor graph using the Louvain algorithm according to the top 30 principal component and cell types, including malignant and immune cells, were assigned according to previous reports (27, 28).

### Analysis of the correlation between PRRS and therapeutic sensitivity

To evaluate the correlation between PRRS and temozolomide sensitivity, the estimated half-maximal inhibitory concentration (IC50) of temozolomide in each sample was estimated by ridge regression using the *pRRophetic* package in R based on the Genomics of Drug Sensitivity in Cancer (GDSC; https://www.cancerrxgene.org) (29).

To evaluate the correlation between PRRS and immunotherapy response, the potential response to anti-PD1 and anti-CTLA4 immunotherapy was predicted using the Submap tool in GenePattern (https://cloud.genepattern.org/gp) with human immunotherapy transcriptome data from a previous study (30). Two immunotherapy cohorts [recurrent glioblastoma patients treated with nivolumab or pembrolizumab, which are both anti-PD1 mAb (31); metastatic urothelial carcinoma patients treated with atezolizumab, which is an anti-PD-L1 mAb (32)] were used to validate the predictive value of PRRS in response to PD-L1 blockade.

### Screening for the potential small molecule compounds synergizing immunotherapy

To further unravel the potential small molecule compounds synergizing immunotherapy based on PRRS, weighted gene co-expression network analysis (WGCNA) was performed to identify the gene modules most associated with PRRS and immuneScore using the *WGCNA* package in R in both TCGA and CGGA cohorts (33). Then, DEGs between the high- and low-PRRS groups were identified using the *limma* package in R (P< 0.05 and |FC| > 1.5). Overlapping genes between the module genes and DEGs were confirmed and imported into the STRING database for functional enrichment. Connectivity map (CMap) (https://clue.io/) is a well-established tool to predict potential therapeutic drug candidates for specific genomic perturbation by comparing disease-specific gene signatures with drug-specific gene expression profiles (34, 35). Based on a gene expression matrix of DEGs between the high- and low-PRRS groups, potential small-molecule compounds associated with the expression pattern and their corresponding mechanisms of action were predicted using the CMap database and CMap mode-of-action (MOA) analysis, respectively (35).

### Statistical analysis

All statistical analyses were performed using the R software (version 4.0.2). An unpaired *t*-test was performed to compare two normally distributed variables, whereas the Wilcoxon rank-sum test was performed for non-normally distributed variables. The Kruskal–Wallis test or one-way analysis of variance was performed to compare three or more variables based on the results of the normal distribution criteria test. Spearman’s correlation coefficient was used to determine correlations between variables. The *survminer* package in R was used to compare the survival status between the two groups, with Kaplan–Meier analysis used to plot survival curves. The log-rank test was performed to determine statistical significance, set at P< 0.05 (two-tailed).

## Results

### Expression pattern of pyroptosis-related genes can separate glioma patients into two clusters with distinct survival status

**Figure 1:**
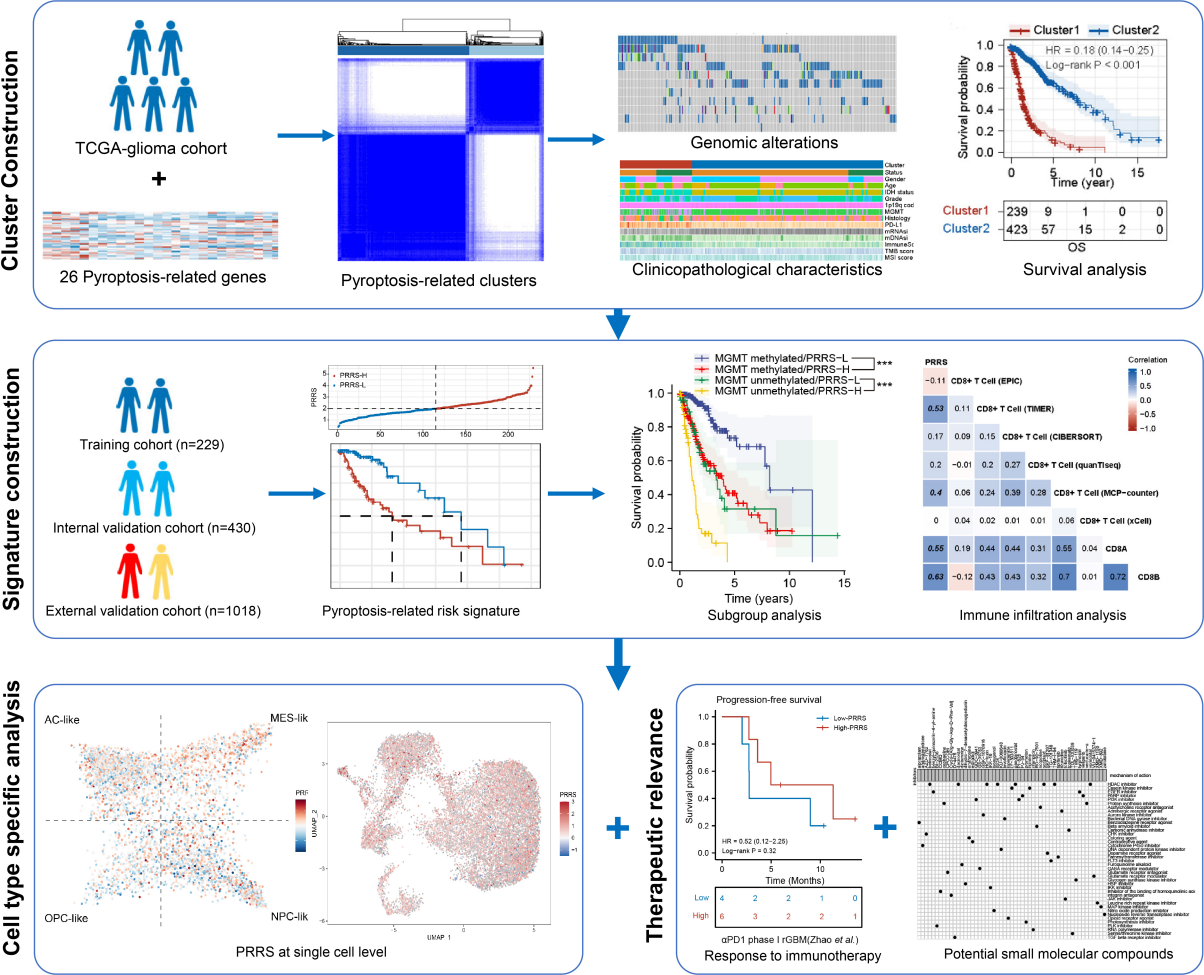
Schematic diagram of working flow.

**Figure 2:**
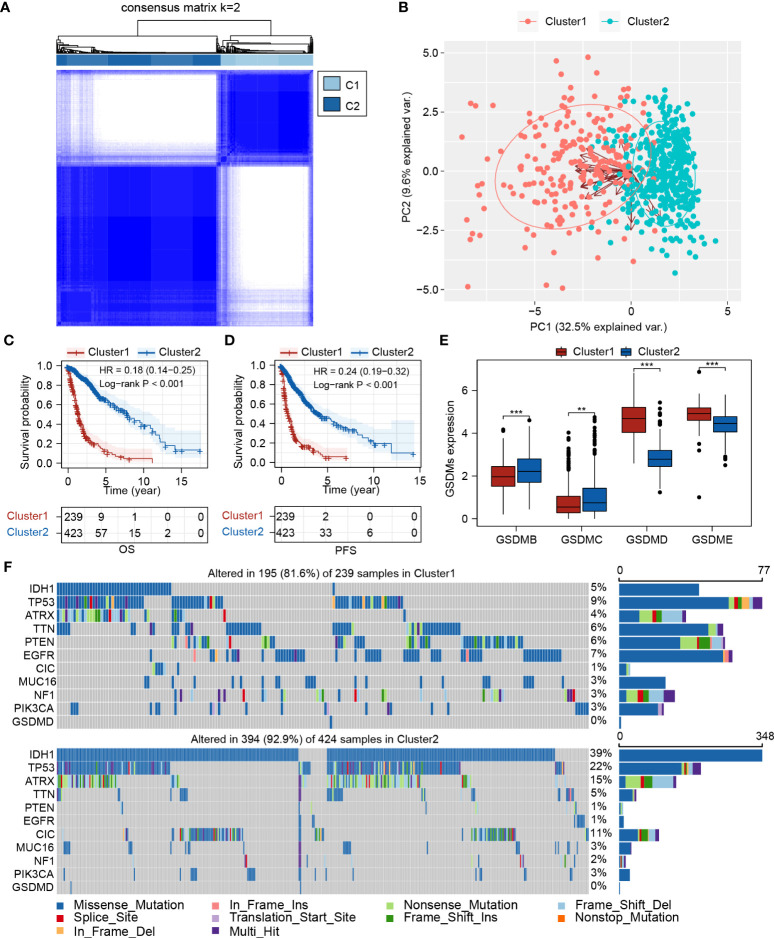
Expression pattern of pyroptosis-related genes could separate glioma patients into two clusters with distinct survival status. **(A)** Consensus clustering of TCGA-glioma cohort (n=662) based on pyroptosis-related genes. **(B)** PCA plot showed the separation between Cluster 1 and Cluster 2. **(C, D)** Survival analysis between Cluster 1 and Cluster 2 for OS and PFS, respectively. **(E)** Expression analysis of *GSDMB*, *GSDMC*, *GSDMD*, and *GSDME* in Cluster 1 and Cluster 2. **(F)** Mutation landscape analysis of Cluster 1 and Cluster 2. PCA, principal component analysis; IDH1, isocitrate dehydrogenase 1; TP53, tumor protein 53; ATRX, ATRX Chromatin Remodeler; TTN, titin; PTEN, phosphatase and tensin homolog; EGFR, epidermal growth factor receptor; CIC, capicua transcriptional repressor; MUC16, mucin 16, cell surface associate; NF1, neurofibromin 1; PIK3CA, phosphatidylinositol-4,5-bisphosphate 3-kinase catalytic subunit alpha; GSDMD, Gasdermin D. **P < 0.005; ***P < 0.001.

A schematic of the working flow is shown in **Figure 1**. Using the 26 pyroptosis-related genes (**Supplementary Table 2**), the TCGA-glioma cohort was divided into two clusters by consensus clustering (**Figure 2A**; **Supplementary Table 4**), which showed satisfactory separation based on PCA analysis (**Figure 2B**). Cluster 2 showed significantly longer overall survival (OS) (HR = 0.18, 95% CI = 0.14–0.25, P< 0.001) and progression-free survival (PFS) (HR = 0.24, 95% CI = 0.19–0.32, P< 0.001) than Cluster 1 (**Figures 2C, D**). Within the GSDM family, *GSDMB*, *GSDMC*, *GSDMD*, and *GSDME* have been well-studied and are known downstream activating enzymes involved in pyroptosis (36). Therefore, the expression patterns of these four genes were examined in two clusters. While upregulated expression of *GSDMB* and *GSDMC* was found in Cluster 2 compared to that in Cluster 1, Cluster 1 had higher *GSDMD* and *GSDME* expression levels than Cluster 2, indicating that different pyroptosis pathways may be activated in different pyroptosis-related clusters (**Figure 2E**). Gene mutations are major drivers of tumorigenesis and tumor progression (37, 38). Thus, mutations in several driver genes in gliomas were assessed (**Figure 2F**). While 39% of the patients in Cluster 2 were found to possess IDH1 mutations, only 5% of the patients in Cluster 1 had IDH1 mutations (**Figure 2F**). Moreover, phosphatase and tensin homolog deleted on chromosome ten (PTEN), a tumor suppressor previously reported to promote pyroptosis by enabling NLRP3/ASC1 assembly *via* NLRP3 dephosphorylation (39), was found to be more frequently mutated in Cluster 1 (**Figure 2F**). In addition, a higher missense mutation rate of the Drosophila gene capicua (CIC) was found in Cluster 2 (**Figure 2F**), which has been shown to correlate with better survival in glioma patients, even with the co-occurrence of favorable markers including IDH mutation and 1p/19q codeletion (40).

### The clinical characteristics and immune infiltration status differ between Cluster 1 and Cluster 2

**Figure 3:**
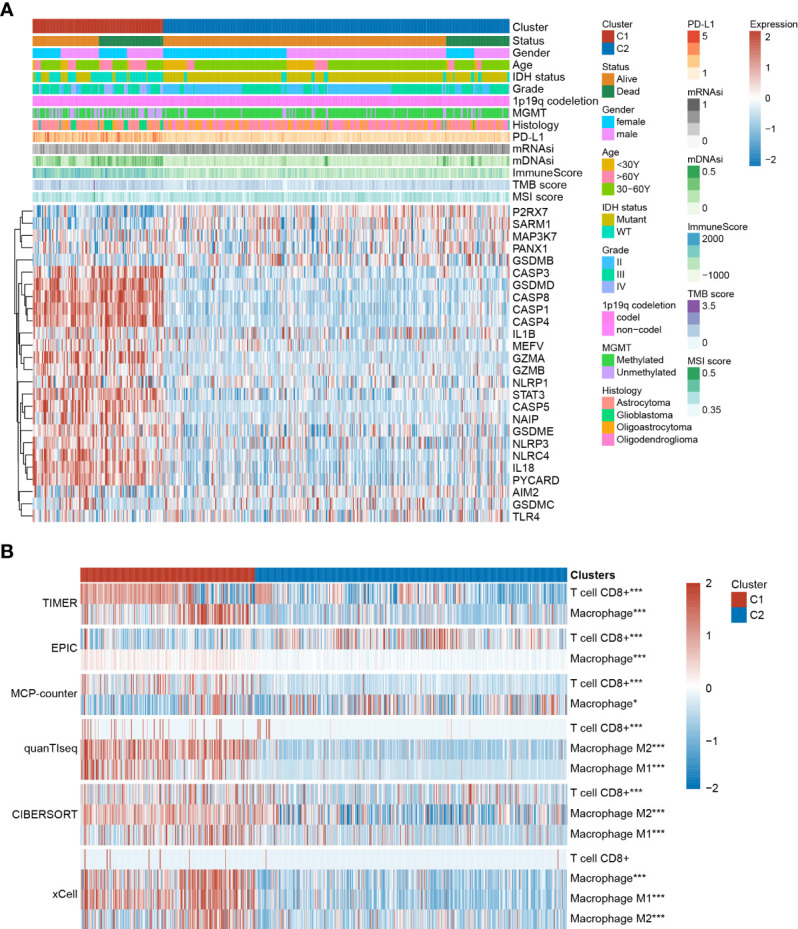
The clinical characteristics and immune infiltration status differ between Cluster 1 and Cluster 2. **(A)** Clinical and molecular characteristics of Cluster 1 and Cluster 2. **(B)** Immune infiltration analysis based on TIMER, EPIC, MCP-counter, quanTIseq, CIBERSORT, and xCell algorithms. *P < 0.05; **P < 0.005; ***P < 0.001.

We further characterized the clinical and molecular characteristics of Clusters 1 and 2. The pyroptosis-related genes showed distinct expression patterns between Cluster 1 and Cluster 2, with significantly higher expression levels of *GSDMD*, *CASP1*, *CASP3*, *CASP4*, and *CASP8*, and lower expression levels of *P2RX7* and *SAMR1* observed in Cluster 1. Cluster 1 was mainly composed of glioblastomas and grade III gliomas, and Cluster 2 was mainly composed of grade II gliomas (**Figure 3A**). Consistently, IDH1 mutations and MGMT promoter methylation were more frequent in Cluster 2 (**Figure 3A**). The mDNAsi index (which reflects epigenetic stemness features) and the mRNAsi index (which reflects transcriptomic stemness features) were calculated, as previously described (24). Although Cluster 1 possessed a lower mRNAsi index, contradictory results were found for the mDNAsi index. This indicates that diverse stemness regulation processes should be focused upon in Clusters 1 and 2 (**Figure 3A**; **Supplementary Figure 1**). A higher ImmuneScore and increased PD-L1 and CTLA-4 expression levels were identified in Cluster 1 (**Supplementary Figure 1**), suggesting a more immunosuppressive tumor ecosystem. Furthermore, higher TMB and lower MSI scores were observed in Cluster 1 (**Figure 3A**; **Supplementary Figure 1**; **Supplementary Table 5**).

Immune infiltration of different clusters was evaluated using six commonly used algorithms. It has been previously reported that tumor-associated macrophages (TAMs) and CD8^+^ T cells were considered the predominant immune cells involved in glioma progression. Accordingly, most algorithms showed higher CD8+ T cell enrichment in Cluster 1. Additionally, higher infiltration of both M1 and M2 macrophages was observed, especially in the latter (**Figure 3B**). GSEA results showed that in several immune-related pathways (including the IL-2, IL-6, IFN-γ, and TNF-α pathways) and the inflammatory response were highly enriched in Cluster 1 compared to in Cluster 2 (**Supplementary Figure 1**), indicating the regulatory role of these pathways in modulating cluster-specific immune microenvironments.

### Establishment of the pyroptosis-related risk signature

Pyroptosis-related clusters established with pyroptosis-related genes focused on the gene co-expression pattern but were also prognostic for glioma patients. To further confirm the prognostic value of the pyroptosis-related signature and establish a scoring system, the TCGA glioma cohort was divided into a training cohort (n=229) and an internal validation cohort (n=430), and a scoring system, called pyroptosis-related risk signature, based on 26 pyroptosis-related genes was then established by univariate and multivariate Cox regression analysis (**Supplementary Figure 2**). Eight pyroptosis-related genes were identified, and the final formula for calculating PRRS was as follows:


$$\begin{array}{l} PRRS=(0.9548\times CASP5\;expression\;level)+(-0.4692\times \\ GSDMB\;expression\;level)+(0.1130\times GZMA\;expression\;level)+\\ (0.5692\times GZMB\;expression\;level)+(1.665\times MEFV\;expression\\ level)+(-0.8230\times NLRC4\;expression\;level)+\\ (-0.6217\times SARM1\;expression\;level)+(0.8052\times \\ STAT3\;expression\;level) \end{array}$$

**Figure 4:**
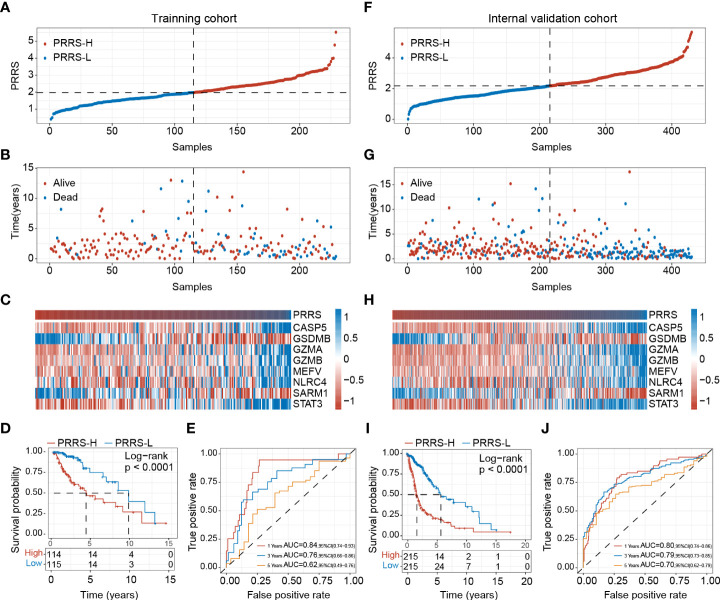
Establishment of pyroptosis-related risk score (PRRS). **(A)** PRRS value in the training cohort. **(B)** Survival status in the training cohort. **(C)** Expression pattern of the PRRS genes in the training cohort. **(D)** Survival analysis of different risk group for overall survival in the training cohort. **(E)** ROC curve analysis for 1-year, 3-year and 5-year overall survival in the training cohort. **(F)** PRRS value in the internal validation cohort. **(G)** Survival status in the internal validation cohort. **(H)** Expression pattern of the PRRS genes in the internal validation cohort. **(I)** Survival analysis of different risk group for overall survival in the internal validation cohort. **(J)** ROC curve analysis for 1-year, 3-year and 5-year overall survival in the internal validation cohort.

In the training cohort, patients were divided into low- and high-PRRS groups based on the median value (**Figure 4A**); more alive-status samples were in the low-PRRS group (**Figure 4B**). The high-PRRS group had higher expression levels of *CASP5*, *GZMA*, *GZMB*, *MEFV*, *NLRC4*, and *STAT3* than the low-PRRS group, whereas higher *GSDMB* and *SARM1* expression levels were observed in the low-risk group (**Figure 4C**). PRRS can satisfactorily divide glioma patients into two groups with distinct survival statuses. Specifically, the low-PRRS group had a significantly longer OS than the high-PRRS group (median OS: 9.506 and 4.085 years in the low-PRRS and high-PRRS groups, respectively; **Figure 4D**), and PRRS also showed satisfactory performance for 1-year, 3-year and 5-year OS predictions (1-year AUC = 0.84, 3-year AUC = 0.76, and 5-year AUC = 0.62; **Figure 4E**). In the internal validation cohort, PRRS showed a similar prognostic value for glioma patients (median OS: 5.622 and 1.537 years in the low- and high-risk groups, respectively; HR = 3.576, 95% CI = 2.672–4.789, P<0.001; **Figure 4F–I**). As in the training cohort, the AUC for the 1-year, 3-year and 5-year OS were 0.80, 0.79, and 0.70, respectively (**Figure 4J**).

The CGGA-325 and CGGA-693 cohorts were further used for external validation, and PRRS showed robust performance for prognosis prediction in these two cohorts (**Supplementary Figures 3, 4**). In CGGA-325 cohort, the low-PRRS group had a significantly longer OS compared with the high-PRRS group (median OS: 6.1 and 1.2 years in the low-PRRS and high-PRRS groups, respectively; HR = 2.20, 95% CI = 1.67–2.89, P<0.001; **Supplementary Figure 3**). PRRS also showed satisfactory prediction performance in the CGGA-693 cohort (median OS: 6.8 and 1.7 years in the low-risk and high-risk groups, respectively; HR = 2.20, 95% CI = 1.80–2.69, P<0.001; **Supplementary Figure 4**). Collectively, PRRS is an ideal tool for predicting the prognosis of patients with glioma.

### The prognostic value of PRRS remains under a subgroup analysis

**Figure 5:**
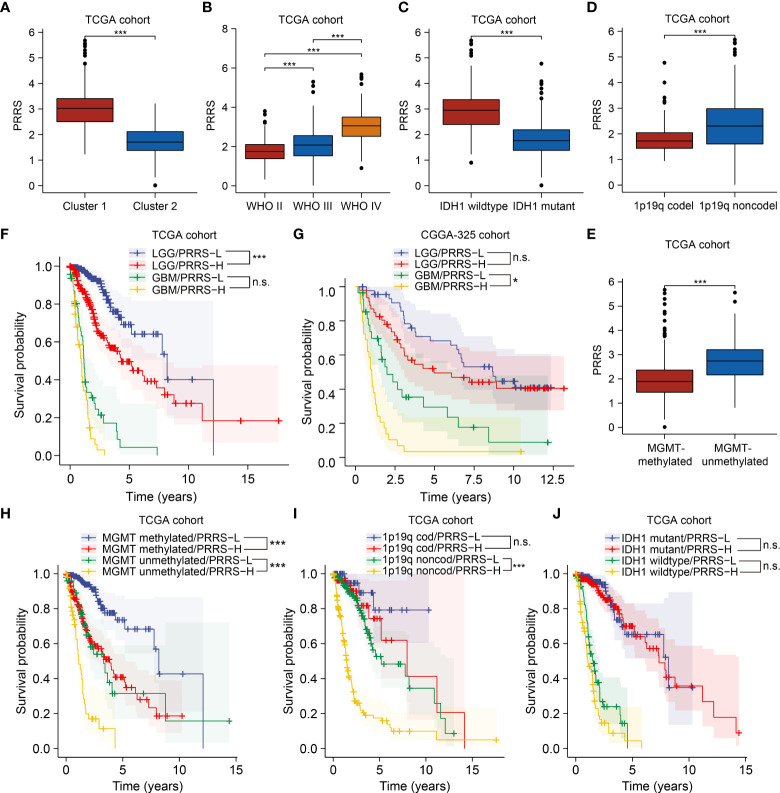
The prognostic value of PRRS remains under a subgroup analysis. **(A)** Comparison of PRRS in Cluster 1 and Cluster 2 in TCGA cohort. **(B)** Comparison of PRRS in WHO grade II, grade III, and grade IV gliomas in TCGA cohort. **(C)** Comparison of PRRS in IDH1 wildtype and mutant gliomas in TCGA cohort. **(D)** Comparison of PRRS in 1p/19q codeleted and non-codeleted gliomas in TCGA cohort. **(E)** Comparison of PRRS in MGMT promoter methylated and unmethylated gliomas in TCGA cohort. **(F)** Survival analysis of glioma patients with low and high PRRS in LGG and GBM group, respectively, in TCGA cohort. **(G)** Survival analysis of glioma patients with low and high PRRS in LGG and GBM group, respectively, in CGGA-325 cohort. **(H)** Survival analysis of glioma patients with low and high PRRS in co-occurrence with MGMT promoter methylation or not in TCGA cohort. **(I)** Survival analysis of glioma patients with low and high PRRS in co-occurrence with 1p/19q codeletion or not in TCGA cohort in TCGA cohort. **(J)** Survival analysis of glioma patients with low and high PRRS in co-occurrence with IDH1 mutation or not in TCGA cohort. *P < 0.05; **P < 0.005; ***P < 0.001, n.s., not significant.

PRRS was evaluated in different clinical subgroups. High PRRS was observed in Cluster 1 compared to that in Cluster 2, in accordance with the poorer prognosis found in Cluster 1 (**Figure 5A**). While PRRS increased as the stage advanced (**Figure 5B**), higher PRRS was also observed in IDH1 wildtype, 1p/19q non-codeleted, and MGMT-promoter unmethylated gliomas compared to their counterparts (**Figure 5C-E**). Since lower grade, 1p/19q codeletion, MGMT promoter methylation, and IDH1 mutation were the major favorable prognostic markers for glioma, a subgroup analysis based on the status of these parameters was performed in both TCGA and CGGA cohort.

PRRS showed satisfactory stratification for prognosis in the TCGA-LGG cohort (HR = 2.39, 95% CI = 1.56-3.66, adjusted P-value = 0.001). GBM patients with low PRRS also showed longer survival times than those with high PRRS, although this difference was not statistically significant (HR = 1.66, 95% CI = 1.06-2.60, adjusted P-value = 0.111; **Figure 5F**). Nevertheless, in the CGGA-325 cohort, PRRS was prognostic in the GBM subgroup (for the LGG subgroup, HR = 1.33, 95% CI = 0.75-2.34, adjusted P-value = 1.000; for the GBM subgroup, HR = 2.34, 95% CI = 1.31-4.20, adjusted P-value = 0.012; **Figure 5G**), whereas in the CGGA-693 cohort, PRRS also showed an association with decreased overall survival time in both LGG and GBM subgroups, although the difference was not statistically significant (for the LGG subgroup, HR = 2.19, 95% CI = 1.55-3.09, adjusted P-value<0.001; for the GBM subgroup, HR = 1.15, 95% CI = 0.73-1.82, adjusted P-value = 1.000; **Supplementary Figure 5**). To further consolidate this result, we obtained the expression and survival data from another GBM cohort from CPTAC and found similar results (HR = 1.57, 95% CI = 0.94-2.62, P value = 0.088; **Supplementary Figure 5**; **Supplementary Table 6**).

In both TCGA and CGGA cohorts, high PRRS was significantly associated with poor prognosis in both MGMT methylated and unmethylated subgroups (**Figure 5H**; **Supplementary Figure 5**). Considering the 1p/19q codeletion status, in TCGA and CGGA-325 cohorts, low PRRS predicted longer OS time in glioma patients without 1p/19q codeletion, whereas in the CGGA-693 cohort, PRRS was a significantly prognostic predictor in patients with or without 1p/19q codeletion (**Figure 5I**; **Supplementary Figure 5**). Further investigation found that PRRS could not be a significant prognostic predictor in either the IDH1 wildtype or mutant subgroups in the TCGA cohort, although high PRRS was still correlated with unfavorable survival in both subgroups (**Figure 5J**). Nevertheless, high PRRS is still a valuable marker for poorer prognosis in the IDH1 wild-type subgroup in the CGGA-325 cohort and in the IDH1 mutant subgroup in the CGGA-693 subgroup (**Supplementary Figure 5**; **Supplementary Table 6**).

### PRRS is positively correlated with CD8+ T cell infiltration in glioma

**Figure 6:**
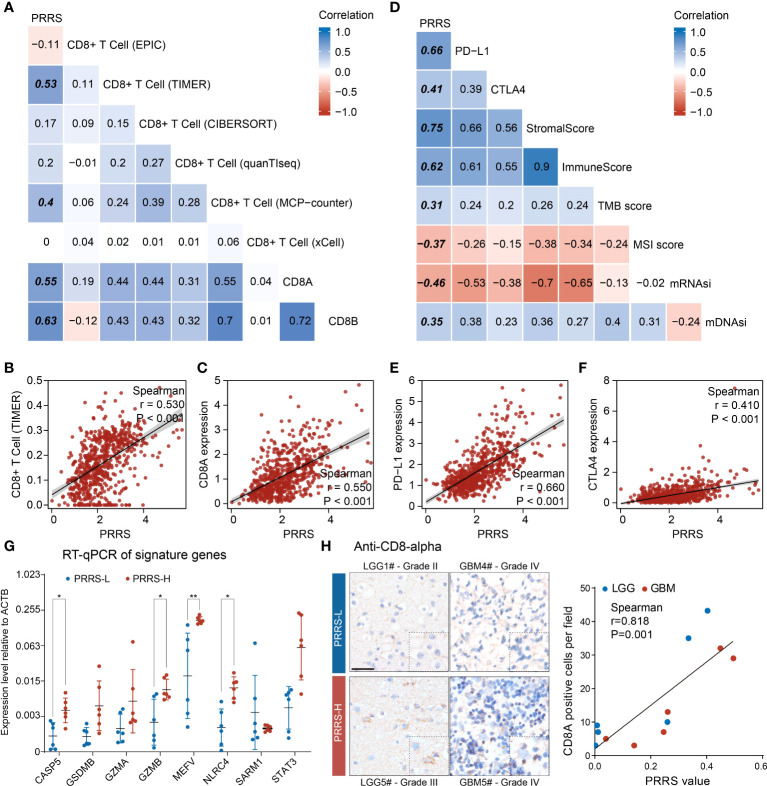
PRRS is correlated with CD8+ T cell infiltration in glioma. **(A, B)** The correlation between PRRS and CD8+ T cell was analyzed using EPIC, TIMER, CIBERSORT, quanTIseq, MCP-counter, and xCell algorithms. **(C)** Dot graph showed the correlation between PRRS and CD8A expression. **(D)** The correlation between PRRS and PD-L1, CTLA-4, Stromal score, Immune score, TMB score, MSI score, mRNAsi, and mDNAsi was analyzed. **(E, F)** Dot graph showed the correlation between PRRS and PD-L1 or CTLA-4 expression. **(G)** RT-qPCR analysis of signature genes in twelve glioma tissues. **(H)** The infiltration of CD8+ T cell was examined with anti-CD8-α staining in low- and high-PRRS glioma tissues, respectively, and correlation plot and Spearman’s correlation coefficient analysis were performed. Scale bar = 50 µm. *P < 0.05; **P < 0.005.

We previously found that different pyroptosis-related clusters were associated with distinct immune infiltration. To determine the potential value of PRRS in immunotherapy, we first evaluated the correlation between PRRS and CD8+ T cells, which are canonical effector cells in immunotherapy (**Figure 6A**). Among the six commonly used algorithms, PRRS was significantly correlated with CD8+ T-cell enrichment in the TIMER and MCP-counter algorithms (r = 0.530, P<0.001 for the TIMER algorithm; r = 0.400, P<0.001 for the MCP-counter algorithm; **Figure 6B**; **Supplementary Figure 6**). To further validate this result, the correlation between PRRS and *CD8A* and *CD8B* was analyzed, and a significant positive correlation between PRRS and *CD8A* and *CD8B* was confirmed (r = 0.550, P<0.001 for *CD8A*; r = 0.630, P<0.001 for *CD8B*; **Figure 6C**; **Supplementary Figure 6**). Consistently, PRRS was positively correlated with both stromal and immune scores (r = 0.750, P<0.001 for stromal score; r = 0.620, P<0.001 for immune score; **Figure 6D**; **Supplementary Figure 6**). However, a positive correlation between PRRS and immune checkpoints, including PD-L1 and CTLA-4, was also found (r = 0.660, P<0.001 for PD-L1; r = 0.410, P<0.001 for CTLA-4; **Figure 6E, F**), indicating that although a high PRRS was associated with more CD8+ T cell infiltration, the infiltrated T cells may be exhausted because of the concomitantly high expression of immune checkpoints. While PRRS was positively correlated with TMB score, it was negatively associated with MSI score (r = 0.310, P<0.001 for TMB score; r = -0.370, P<0.001 for MSI score; **Supplementary Figure 6**). Consistently, in both CGGA-325 and CGGA-693 cohorts, PRRS was positively correlated with CD8+ T cell enrichment and higher immune checkpoint expression levels (**Supplementary Figure 7**).

We subsequently validated the relationship between PRRS and CD8+ T cell infiltration in glioma samples. Through quantification of signature genes by RT-qPCR and calculation of PRRS according to the formula mentioned above, 12 glioma samples were divided into low- and high-PRRS groups. Higher expression of CASP5, GZMB, MEFV, and NLRC4 was found in the high-PRRS group (**Figure 6G**; **Supplementary Table 7**). CD8+ T cell infiltration was examined in the low- and high-PRRS groups using IHC. It was previously reported that T-cell infiltration can be highly variable in gliomas (41), and we observed that LGG or GBM samples with high PRRS harbored more CD8+ T cells than those with low PRRS (**Figure 6H**). Moreover, a positive correlation was observed between PRRS and CD8+ T-cell enrichment (**Figure 6H**).

### PRRS genes are expressed at a cell type-specific manner

**Figure 7:**
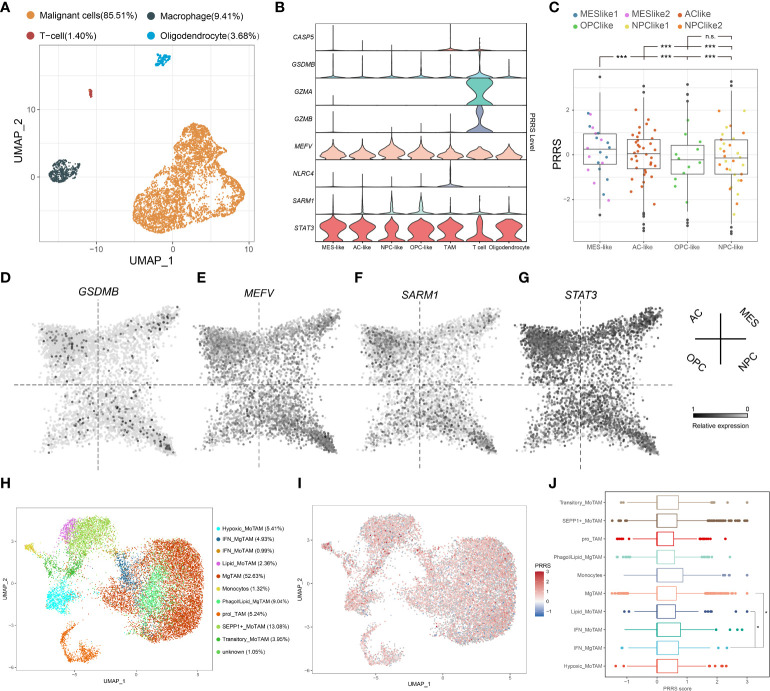
PRRS genes are expressed at a cell type-specific manner. **(A)** UMAP plot of malignant cells and immune cells was performed on TPM-normalized data. **(B)** Expression pattern of PRRS gene in different cell type. **(C)** PRRS value in different subtype of GBM cells. **(D-G)** Expression pattern of *GSDMB*, *MEFV*, *SARM1*, and *STAT3* in different subtype of GBM cells. **(H)** UMAP plot of TAMs was performed on RPCA-based integrated data. **(I)** PRRS value was calculated in different subtype of TAMs. TAMs, tumor-associated macrophages/microglia. MoTAM, monocyte-derived TAM; MgTAM, microglia-derived TAM. *P < 0.05; ***P < 0.001, n.s., not significant.

*GZMA* and *GZMB* were previously reported to be specifically expressed in CD8+ T cells (42), indicating that PRRS is a signature involving not only tumor cells but also immune cells. To further illustrate the potential cell type-specific expression pattern, the expression of eight PRRS genes in glioblastoma cells, TAMs, T cells, and oligodendrocytes was analyzed using scRNA data obtained in a previous study (**Figure 7A**) (27). *STAT3* and *MEFV* are ubiquitously expressed in several cell types. Conversely, higher expression levels of *GSDMB*, *GZMA*, and *GZMB* were detected in T cells, whereas *NLR4* and *CASP5* showed specifically high expression levels in TAMs, and *CASP5*, *GZMA*, *GZMB*, and *NLRC4* were barely detected in glioblastoma cells (**Figure 7B**). Neftel *et al.* stratified malignant glioblastoma cells into four cellular states: neural-progenitor-like (NPC-like), oligodendrocyte-progenitor-like (OPC-like), astrocyte-like (AC-like), and mesenchymal-like (MES-like) states (27). It has been suggested that glioblastoma cells in the MES-like state may be more efficiently killed by T cells (43). By calculating the PRRS in each cellular state, we found that MES-like and AC-like glioblastoma cells possessed a higher PRRS score than those in the other two states (**Figure 7C**). Specifically, while *MEFV* and *STAT3* expression was detected in both glioblastoma cells and immune cells, *SARM1* expression level was higher in NPC-like and OPC-like glioblastoma cells than in MES-like, AC-like glioblastoma cells, and other tumor cells (**Figure 7D–G**). Antunes *et al.* divided myeloid cells in GBM into several subsets (28). PRRS was calculated for each type of TAMs. A previous study suggested that higher interferon γ (IFNγ) response was associated with immunotherapy response in GBM, and IFNγ could modulate the immune cell composition in the tumor center (44). Intriguingly, a significantly higher PRRS was found in interferon-signature microglial-TAM (IFN_MgTAM) than in non-interferon-signature microglial-TAM (MgTAM) (**Figure 7H, I**).

### Pyroptosis-related cluster and PRRS are associated with survival benefits from temozolomide and immunotherapy

**Figure 8:**
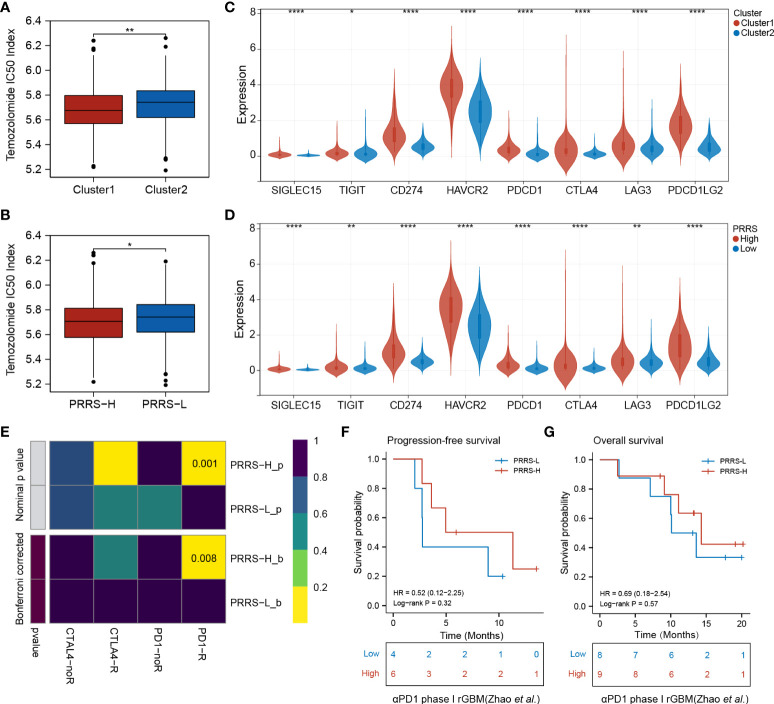
Pyroptosis-related cluster and PRRS are associated with survival benefits from temozolomide and immunotherapy. **(A)** Predicted TMZ sensitivity in Cluster 1 and Cluster 2. **(B)** Predicted TMZ sensitivity in high- and low-PRRS group. **(C)** Expression levels of immune checkpoints in Cluster 1 and Cluster 2. **(D)** Expression levels of immune checkpoints in high- and low-PRRS group. **(E)** Submap analysis of the predicted response to anti-CTLA4 and anti-PD1 in high- and low-PRRS group. **(F)** Survival analysis for progression-free survival between low- and high-PRRS group. **(G)** Survival analysis for overall survival between low- and high-PRRS group. *P < 0.05; **P < 0.005; ****P < 0.0001.

Based on the prognostic value of the pyroptosis-related group and PRRS, and the distinct tumor-associated immune microenvironment between different groups, further investigation into their roles in therapeutic response was performed. Both Cluster 1 and the high-PRRS group had a lower temozolomide IC50 index than their counterparts, indicating that Cluster 1 and the high-PRRS group were more sensitive to TMZ therapy (**Figure 8A, B**). Higher expression levels of several immune checkpoints were also observed in Cluster 1 and the high PRRS group (**Figure 8C, D**, **Supplementary Table 8**). Subsequently, a submap algorithm was used to evaluate the potential value of PRRS in predicting immunotherapy responses. The results showed that glioma patients with high PRRS might be more responsive to anti-PD1 therapy (nominal P-value = 0.001 and Bonferroni corrected P-value = 0.008; **Figure 8E**). A recurrent glioblastoma (rGBM) cohort that received anti-PD1 mAb therapy was used for validation (31). Both PFS and OS of GBM patients with high PRRS showed a trend toward improved response compared with the low-PRRS group. However, these differences did not reach statistical significance, partially due to the limited sample size (**Figure 8F, G**). In the Imvigor210 cohort, a clinical trial involving urothelial carcinoma patients who received anti-PD-L1 therapy, a survival benefit was not observed. However, a higher percentage of inflamed immune phenotype, which is considered a “hot” tumor, was observed in the high-PRRS group, indicating that PRRS could stratify patients into different immune phenotypes (**Supplementary Figure 8**).

### Identification of small-molecule compounds to synergize immunotherapy

**Figure 9:**
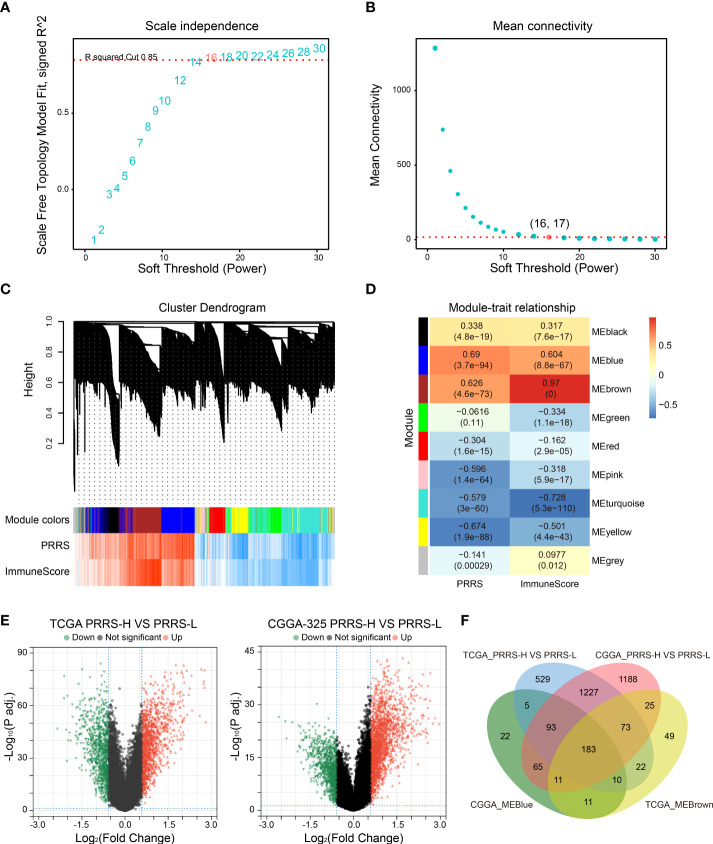
Identification of canonical module genes related with PRRS by weighted gene co-expression network analysis (WGCNA). **(A, B)** Determination of soft-threshold power in WGCNA. The most appropriate power was 16 **(A)**, and the corresponding number of mean connectivity was 17 **(B)**. **(C)** Cluster dendrogram and module assignment in WGCNA. **(D)** Association between module and PRRS and ImmuneScore through module–trait relationship analysis. **(E)** Differentially expressed genes (DEGs) were analyzed between high- and low-PRRS groups in TCGA and CGGA cohort, respectively. **(F)** Venn diagram showing the overlap between module genes and DEGs.

**Figure 10:**
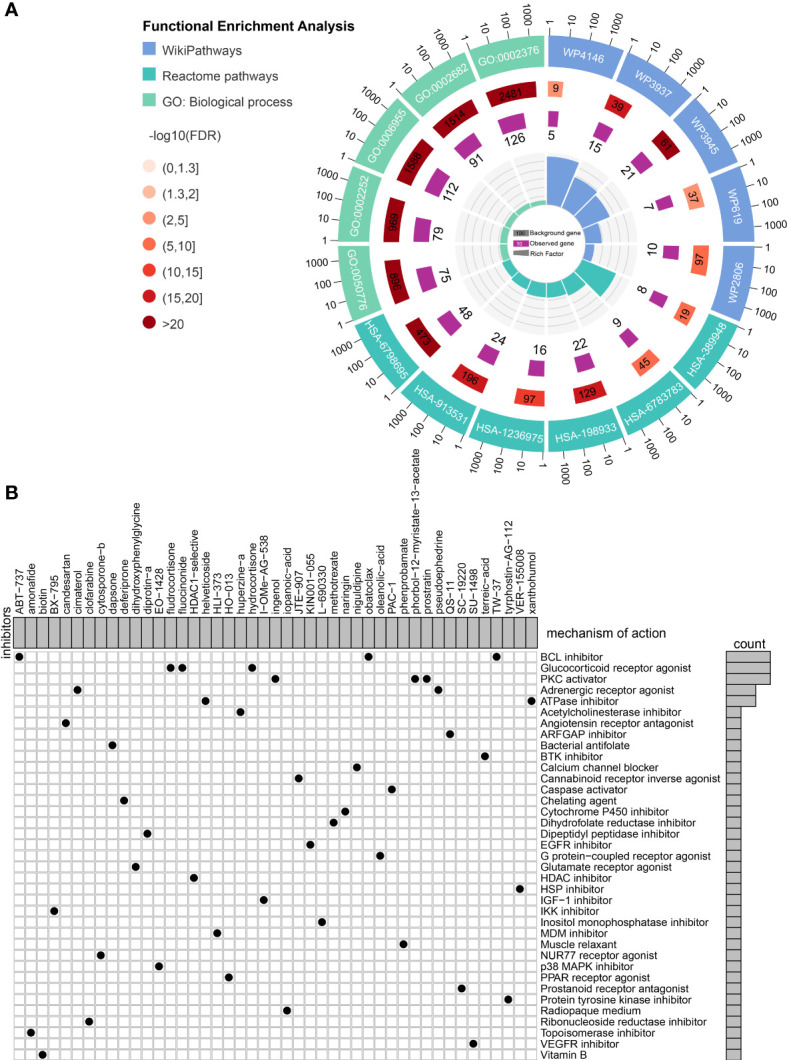
Identification of small molecule compounds to synergize with immunotherapy. **(A)** Functional enrichment of the overlapped genes between module genes and differentially expressed genes. **(B)** Mechanism of action analysis of the identified small molecule compounds.

Connectivity map (CMap) is a well-established tool for predicting potential small-molecule compounds for particular genomic perturbations or diseases by comparing specific gene signatures with drug-specific gene expression profiles in the reference database (35). To further deepen the therapeutic value of PRRS, subsequent screening using CMap was performed for potential therapeutic drug candidates associated with PRRS-related expression patterns. First, WGCNA was performed in the TCGA cohort, and nine co-expression gene modules were identified based on a power of 16 (**Figure 9A**), while the number of mean connectivity was 17 (**Figure 9B**), including black, blue, brown, green, red, pink, turquoise, yellow, and gray; the gray module was considered to be a group of genes that could not be assigned to any module (**Figure 9C**, **Supplementary Table 9**). Among these nine modules, the brown module showed the most positive correlation between both PRRS and ImmuneScore (**Figure 9D**). Using the same methodology, the blue module, which was highly associated with PRRS and ImmuneScore, was identified in the CGGA-325 cohort (**Supplementary Figure 9**, **Supplementary Table 10**). Through further analysis of DEGs, we identified 2142 and 2865 DEGs between the high- and low-PRRS groups in TCGA and CCGA-325 cohorts, respectively (**Supplementary Table 11**–**12**; **Figure 9E**). The intersection of genes from the TCGA brown module, the CGGA blue module, and DEGs created a list of 183 genes (**Figure 9F**). Subsequent functional enrichment analysis showed significant enrichment in immune response, immune system process, immune effector process, and regulation of immune response for biological process analysis. For the REACTOME pathway analysis, neutrophil degranulation, interferon signaling, antigen processing-cross presentation, and PD-1 signaling were enriched. For the wikiPathway analysis, the microglia pathogen phagocytosis pathway, complement system, macrophage marker, and type II interferon signaling pathways were enriched (**Figure 10A**). Ultimately, based on 183 upregulated genes and 63 downregulated genes in the high-PRRS group compared to in the low-PRRS group, potential small molecular compounds and their related mechanisms were predicted by the CMap database and MOA analysis, respectively (**Figure 10B**, **Supplementary Table 13**). These results indicate that treatment with targeted drugs, such as Bcl-2 and ATPase inhibitors, may facilitate immunotherapy in gliomas, although further evidence is required.

## Discussion

Pyroptosis is a specific form of programmed cell death, characterized by an inflammatory response that is distinct from apoptosis (9). Pyroptosis can be activated *via* canonical or noncanonical pathways. Canonical pyroptotic death is mediated by inflammasome assembly, consisting of (i) inflammasome-forming sensors, including NOD-like receptors (NLRs), proteins absent in melanoma 2 (AIM2) and pyrin (protein coded by *MEFV*), (ii) adapter apoptosis-associated speck-like protein containing a caspase recruitment domain (CARD) (ASC), and (iii) procaspase-1 (45). Unlike inflammasomes activated by NLRPs (such as NLRP3 and NLRP1), the NLRC4-inflammasome consists of two NLR family members, NLRC4 and NAIP (NLR family of apoptosis inhibitory protein) (46). The canonical inflammasome then further cleaves the gasdermin protein, the executioner of pyroptosis, and pro-IL-1β and pro-IL-18, which trigger pyroptosis and the release of mature cytokines through the N-terminal pore-forming domain of cleaved gasdermin members (47). Unlike canonical pyroptosis, the non-canonical pyroptosis pathway has not been extensively studied. Independent of the upstream inflammasome sensory complex, caspase-4/5 can be directly activated by binding to intracellular lipopolysaccharides through their CARD at the N-terminus (6). Activated caspase-4/5 can also cleave gasdermin D (GSDMD) into N-GSDMD, which ultimately forms plasma membrane pores in target cells and leads to pyroptosis (48, 49). Although caspase-8 is well recognized as an apoptosis executioner, it is also involved in pyroptosis following TNF-α stimulation (7, 50). Moreover, granzyme B (GZMB) released from T cells can cleave GSDME directly or through Caspase-3 in target cells and activate extensive pyroptosis, further promoting antitumor immune response and inhibiting tumor growth (51, 52). Granzyme A (GZMA)-derived natural killer cells and cytotoxic T lymphocytes (CTLs) can also directly cleave GSDMB in high-GSDMB target cells and trigger pyroptosis, which updates the current understanding of cytotoxic lymphocyte killing mechanisms (53). Interestingly, high levels of GSDMB are correlated with a poorer response to HER-2 targeted therapy in breast cancer, and may be associated with the malignant phenotype of gastric cancer (54, 55). The pyroptosis-related risk signature (PRRS) established in the present study involved genes from canonical pathways, such as *MEFV* and *NLRC4*; genes from non-canonical pathways, such as *CASP5*, *GZMA*, and *GZMB*; gene-coded pyroptosis executioner, such as *GSDMB*; and genes involved in pyroptosis regulation, such as *STAT3* and *SARM.* Therefore, this signature may provide a more comprehensive understanding of pyroptosis than previous pyroptosis-related signatures identified in glioma (56–58). Moreover, using single-cell analysis, we confirmed the expression of PRRS genes in a cell type-specific manner, indicating that this signature involved both tumor cells and their associated microenvironment, and deepened the current understanding of the tumor microenvironment from a pyroptosis perspective.

Pyroptosis and related components play crucial roles in tumorigenesis and tumor progression, although their relationship is diverse due to the different genetic natures of different malignancies. In gastric cancer, GSDMB is highly expressed in most cancerous tissue samples but not in most normal gastric samples, and may be associated with increased levels of invasion (55). Zhou *et al.* found that tumor cells overexpressing GSDMB showed obvious pyroptosis characteristics, and interferon-γ and GZMA secreted from lymphocytes further accelerated this process (53). In non-neuroendocrine lung cancer cells treated with etoposide, loss of YAP increases GSDME expression levels, switches the cell death route from apoptosis to pyroptosis, and sensitizes tumor cells to etoposide (59). During the early stage of colorectal cancer, elevated IL-18 secretion facilitated by NLRP1/NLRP3/pyrin could protect against colorectal cancer through the promotion of epithelial barrier regeneration during the early stages of colorectal cancer. Notably, pyroptosis-related genes can also exert their effects in a pyroptosis-independent manner. For example, while the upregulation of GSDMC is cleaved by caspase-8 and induces pyroptosis in MDA-MB-231 and BT549 breast cancer cells (7), knockdown of GSDMC significantly inhibits proliferation and tumorigenesis in colorectal cancer in a pyroptosis-independent manner (60). The inflammasome sensor, pyrin, can promote the integrity of the intestinal barrier and prevent colitis and tumors (61). Suppression of GSDMD expression in gastric cancer can activate the signal transducer and activator of transcription 3 (STAT3) and phosphatidylinositol 3 kinase/protein kinase B (PI3K/PKB) signaling pathways and regulate cell cycle-related proteins to accelerate the S/G2 phase cell transition (62). Although pyroptosis has been less studied in glioma, Liu *et al.* recently found that high GSDMD expression levels were correlated with poor survival and improved TMZ sensitivity in glioblastoma (63).

Programmed cell death is the major regulator of therapeutic resistance. However, the regulatory role of pyroptosis is not well understood. Chemotherapeutic agents can induce caspase-3-dependent GSDME cleavage, ultimately leading to pyroptosis in cells with high GSDME expression levels. However, chemotherapy resistance exists because GSDME expression levels are low in most tumor cell lines, owing to GSDME promoter methylation (64, 65). Additionally, the combination of BRAF and MEK inhibitors was reported to induce GSDME-dependent pyroptosis through caspase-3 and the release of pro-inflammatory factors in melanoma cells, which is associated with an increase in CD4+ T cell and CD8+ T cell infiltration and decreased TAM levels. On the other hand, a loss of pyroptosis was observed in BRAF inhibitor + MEK inhibitor-resistant tumors (66). TMZ is the first-line treatment for glioma. However, over 50% of patients do not respond to TMZ therapy (67). In the present study, although the high-PRRS group was associated with glioma progression and unfavorable prognosis, the patients showed improved sensitivity to temozolomide and immunotherapy. This result is consistent with the findings of a previous study that evaluated the role of gasdermin D (GSDMD) in GBM. While GSDMD is highly expressed in GBM and associated with poor prognosis, temozolomide treatment leads to pyroptosis through the upregulation of GSDMD and increased IL-1β secretion. This indicates that GBM patients with higher GSDMD expression levels may be more sensitive to temozolomide therapy (63). These paradoxical results may be partially due to the double-edged role of pyroptosis, as discussed above. On the one hand, glioma is a highly pro-inflammatory tumor since the abundant secretion of pro-inflammatory factors, such as IL-1β, IL-6, and high mobility group box protein (HMGB1) through the autocrine or paracrine mechanism accelerates tumor growth (68, 69). Chronic inflammation due to pyroptosis may promote glioma progression through multiple mechanisms involving not only tumor cells but also immune and stromal cells residing in the tumor microenvironment. In contrast, several antibiotic chemotherapeutic reagents can promote severe pyroptosis and cell death (7), and pyroptosis-inducing reagents can reverse or partially eliminate chemoresistance in apoptosis-resistant cells and serve as alternatives for malignancy treatment (70). Moreover, acute pyroptosis may remodel the “cold” tumor immune microenvironment to a “hot” microenvironment and activate antitumor immunity (71).

In the TME, inflammatory processes regulated by pyroptosis may mediate the interaction between tumor cells and neighboring immune cells through specific pathways. NLRP3 promotes inflammasome activation and IL-1β secretion in macrophages. Additionally, *Helicobacter pylori* infection enhances NLRP3 expression and subsequent inflammasome activation and IL-1β release in macrophages, which could enhance the tumorigenesis of gastric cancer (72). Moreover, increased IL-1β levels in the stomach epithelium could contribute to the development of gastric cancer by increasing the number of myeloid-derived suppressor cells (73). Abundant TAM (CD68+/CD163+) infiltration was observed in glioma tissues with high GSDMD expression (63). Diminished lung metastasis of melanoma cells was observed in *NLRP3* knockout mice, with increased infiltration of activated NK cells and production of IFN-γ (74). Colitis-associated cancer was promoted in *NLRP3*-knockout mice due to decreased IL-1β and IL-18 levels in hematopoietic cells. However, disease progression was not diminished in *NLRC4*-deletion mice (75). Notably, NLRC4 can promote cytokine and chemokine release in TAMs and amplify protective IFN-γ-producing CD4+ and CD8+ T cells, thereby diminishing tumor growth in melanoma independent of inflammasome assembly (76). In the present study, we established a PRRS with eight pyroptosis-related genes that showed distinct expressions in different cell types. While *STAT3* and *MEFV* were ubiquitously expressed, higher *SARM1* expression levels were observed in GBM cells, higher *NLRC4* expression levels were observed in TAMs, and higher *GSDMB*, *GZMB*, and *GZMA* expression levels were observed in T-cells. This cell type-specific expression pattern may be attributed to specific cellular interactions in the microenvironment. For example, under hypoxia, increased p-STAT3 levels promote the nuclear translocation of PD-L1 in cancer cells, leading to the upregulation of GSDMC transcription. On the other hand, TNF-α derived from macrophages stimulates caspase-8 expression in cancer cells, which further specifically lyses GSDMC into N-GSDMC to induce pyroptosis (7). Moreover, GZMA-derived natural killer cells and CTLs can directly cleave GSDMB and trigger pyroptosis in cancer cells, leading to tumor clearance in a mouse model (53). Therefore, the close interaction between tumor cells and T cells may account for the survival benefit of immunotherapy in the PRRS group, although further validation is needed.

Targeting pyroptosis is emerging as a promising strategy to synergize immunotherapy. ICI therapy elicits durable responses in specific tumor types with highly infiltrated CD8+ T cells. However, most patients do not respond to this therapy. Thus, remodeling the tumor microenvironment to increase CD8+ T cell numbers and ignite the antitumor immune response may be a viable approach to sensitize tumors to immunotherapy (77, 78). For example, Wang *et al.* found that the microbial metabolite trimethylamine N-oxide could induce pyroptosis in breast cancer cells through protein kinase r-like ER kinase and ultimately enhance CD8+ T cell-mediated antitumor immunity (79). Moreover, combined treatment with BRAF and MEK inhibitors induces GSDME-dependent pyroptosis in melanoma cells and subsequently increases the number of intratumoral CD8+ T cells (66). In the present study, based on the pyroptosis-related expression pattern, we screened for potential small inhibitors that may influence pyroptosis, remodeled the tumor immune microenvironment, and identified Bcl-2 or ATPase inhibitors as potential candidates. The BCL-2 inhibitor ABT-737 releases pro-apoptotic BAX protein from Bcl-2 and induces apoptosis in glioblastoma cells both *in vitro* and *in vivo (* 80). However, ABT-737 was less efficient in glioma stem cells with high myeloid cell leukemia 1 (MCL1) expression, and sorafenib targeting MCL1 synergized with ABT-737 to trigger apoptotic cell death in glioma cells (81). To our knowledge, gossypol is the only BCL-2 inhibitor tested in clinical trials for the treatment of recurrent (NCT00540722) and newly diagnosed (NCT00390403) GBM. However, the results of these trials have not yet been published. Obatoclax, a Bcl-2 inhibitor, directly inhibits the proliferation of hepatocellular carcinoma (HCC) cells and sensitizes cancer cells to T cell-mediated killing (82, 83). The combination of obatoclax and anti-PD-1 mAb synergistically inhibited HCC growth in a murine model (83). Similarly, Na+/K+-ATPase inhibition by ouabain and digoxin decreased immune checkpoint expression levels in A549 lung and MDA-MB-231 breast cancer cells, repurposing the currently used reagents to synergize immunotherapy (84). However, it remains unclear whether these inhibitors exert their effects in a pyroptosis-dependent manner.

The pyroptosis-related risk signature established in the present study provides a comprehensive understanding of the potential role of pyroptosis in tumor progression and tumor microenvironment remodeling, and its value in the prediction of survival benefit from immunotherapy was validated in an anti-PD1 cohort. Nevertheless, the present study had several limitations. First, although the risk signature is established based on pyroptosis and provides a comprehensive understanding of pyroptosis-related genes in gliomas, the actual pyroptosis status group in each group should be further confirmed in *in vitro* and *in vivo* experiments since pyroptosis is a complex process involving protein cleavage. Second, we interpreted the potential cell interaction based on the cell-specific expression of pyroptosis-related genes, which requires further validation through co-culture experiments and orthotopic glioblastoma allografts. Third, although we screened several small molecular compounds that synergize cancer immunotherapy, the value and specific mechanism of action require further validation. We expect that the exploration of pyroptosis-related signatures in our study will facilitate further studies on the role of pyroptosis in the TME and immunotherapy.

## Conclusions

In this study, we established a pyroptosis-related risk signature. The high-PRRS group was associated with a poorer prognosis but may be more responsive to TMZ therapy and immunotherapy. While the high PRRS group was correlated with the enrichment of CD8+ T cells, PRRS genes showed a cell type-specific expression pattern, indicating that the potential interaction between tumor cells and immune cells may be involved in pyroptosis. Pyroptosis may be a double-edged sword in glioma owing to its direct effect on tumor cells and indirect effects on the tumor immune microenvironment. Targeting pyroptosis is a promising strategy to optimize glioma immunotherapy.

## Data availability statement

The original contributions presented in the study are included in the article/**Supplementary Material**. Further inquiries can be directed to the corresponding authors.

## Ethics statement

The studies involving human participants were reviewed and approved by Ethics Committees of Nanfang Hospital. The patients/participants provided their written informed consent to participate in this study.

## Author contributions

Research Design: AZ, YS, MC, and YL. Data curation: YZ and YM. Data analysis: YZ, YHC, PC, and MC. Manuscript writing: YZ and YM. Manuscript revision: AZ, YS, MC, YL, YWC, LW, KZ, ZZ, YX, PC, CL, HZ, LZ, XC, and XZ. Supervision: AZ, YS, and MC. All the authors have read and approved the final manuscript. All authors contributed to the article and approved the submitted version.

## Funding

This study was funded by the China Postdoctoral Science Foundation Funded Project (No.2021M701633), the National Natural Science Foundation of China (No.31871406, No. 82102730), Funds for Construction of High-Level Universities in Guangdong Province, and the Natural Science Foundation of Guangdong Province (No.2021A1515011067).

## Acknowledgments

We thank Editage (www.editage.cn) for language polishing.

## Conflict of interest

The authors declare that the research was conducted in the absence of any commercial or financial relationships that could be construed as a potential conflict of interest.

## Publisher’s note

All claims expressed in this article are solely those of the authors and do not necessarily represent those of their affiliated organizations, or those of the publisher, the editors and the reviewers. Any product that may be evaluated in this article, or claim that may be made by its manufacturer, is not guaranteed or endorsed by the publisher.
