# Supplementary material for: Optimization of cancer immunotherapy through pyroptosis: A pyroptosis-related signature predicts survival benefit and potential synergy for immunotherapy in glioma
Source: Front Immunol. 2022 Aug 3;13:961933. doi: 10.3389/fimmu.2022.961933 (PMC9382657; doi:10.3389/fimmu.2022.961933)
Supplement: Supplementary file 1 [file DataSheet_1.pdf]

Optimization of cancer immunotherapy through pyroptosis: a pyroptosis-related signature predicts survival benefit and potential synergy for immunotherapy in glioma

Yu Zeng[1#], Yonghua Cai[2#], Peng Chai[2#], Yangqi Mao[2], Yanwen Chen[1], Li Wang[1], Kunlin Zeng[1], Ziling Zhan[1], Yuxin Xie[1], Peng Chai[1], Cuiying Li[1], Hongchao Zhan[1], Liqian Zhao[1], Xiaoxia Chen[1], Xiaoxia Zhu[3], Yu Liu[4], Ming Chen[5*], Ye Song[2, 6*], Aidong Zhou [1, 3, 7*]

[1] Department of Cell Biology, School of Basic Medical Science, Southern Medical University, Guangzhou, China,

[2] Department of Neurosurgery, Nanfang Hospital, Southern Medical University, Guangzhou, China,

[3] Department of Radiation Oncology, Zhujiang Hospital, Southern Medical University, Guangzhou, China,

[4] Department of Neurosurgery, Shanghai Children's Hospital, Shanghai Jiao Tong University, Shanghai, China,

[5] Department of Neurosurgery, Xinhua Hospital, School of Medicine, Shanghai Jiao Tong University, Shanghai, China,

[6] Department of Neurosurgery, Ganzhou People's Hospital, Ganzhou, China,

[7] Guangdong Province Key Laboratory of Molecular Tumor Pathology, Southern Medical University, Guangzhou, China.

Supplementary Figure 1. Differential analysis of molecular signature between Cluster 1 and Cluster 2. 2

Supplementary Figure 2. Univariate (A) and multivariate cox (B) regression were performed for pyroptosis-related genes. 3

Supplementary Figure 3. Validation of PRRS in CGGA-325 cohort. 4

Supplementary Figure 4. Validation of PRRS in CGGA-693 cohort. 5

Supplementary Figure 5. Subgroup analysis in external validation cohorts. 6

Supplementary Figure 6. The correlation between PRRS and molecular characteristics. 8

Supplementary Figure 7. The correlation between PRRS and immune characteristics in external validation cohorts. 9

Supplementary Figure 8. Validation of PRRS value to predict immunotherapy in IMvigor210. 10

Supplementary Figure 9. Identification of canonical module genes related with PRRS by weighted gene co-expression network analysis (WGCNA) in the CGGA cohort. 11

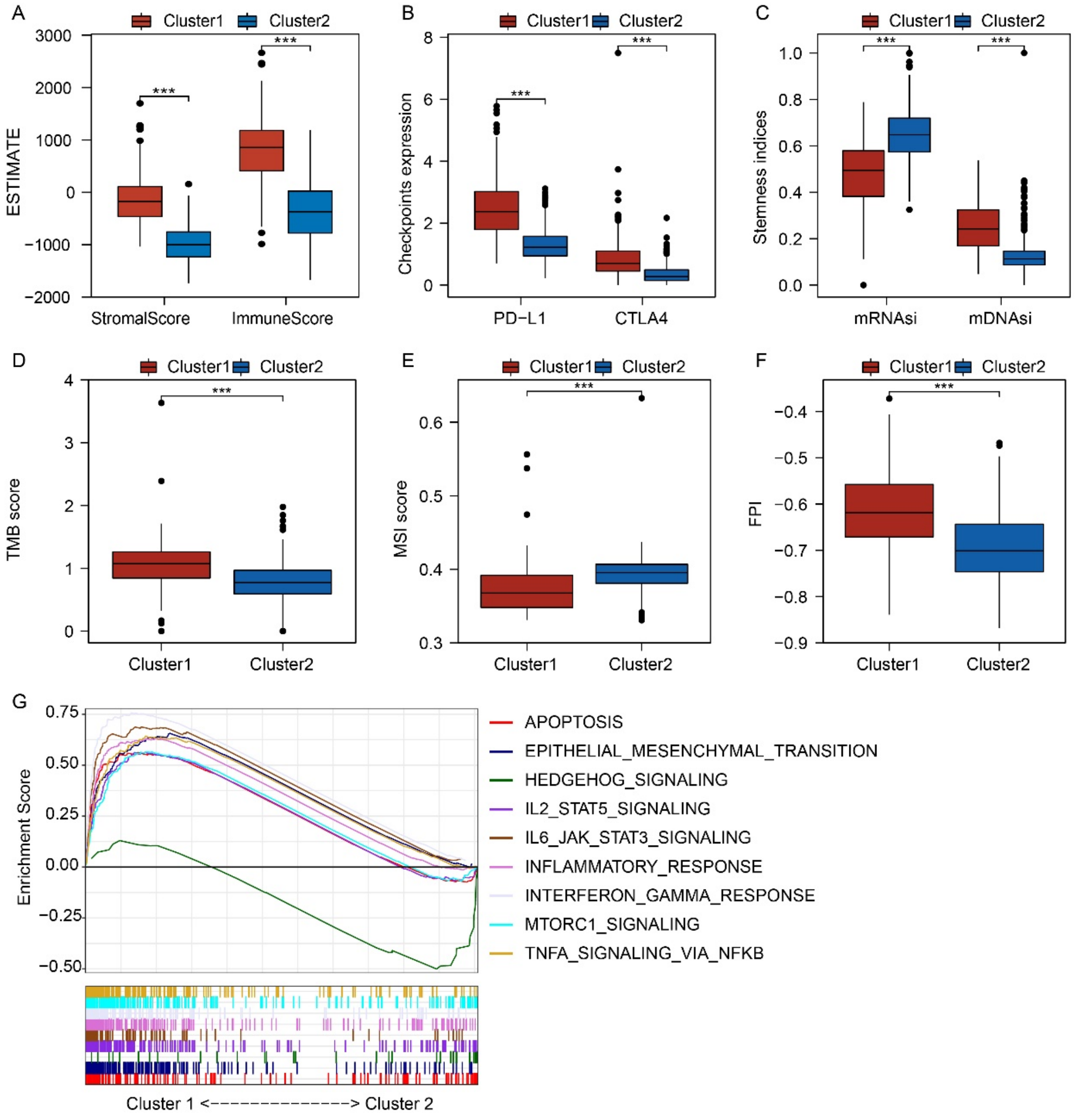

Supplementary Figure 1. Differential analysis of molecular signature between Cluster 1 and Cluster 2. (A-F) Comparison of ESTIMATE score, immune-checkpoint expression level, stemness index, TMB score, MSI score, and FPI between Cluster 1 and Cluster 2. (G) GSEA was performed with the differentially expressed genes between Cluster 1 and Cluster 2. FPI, ferroptosis potential index.

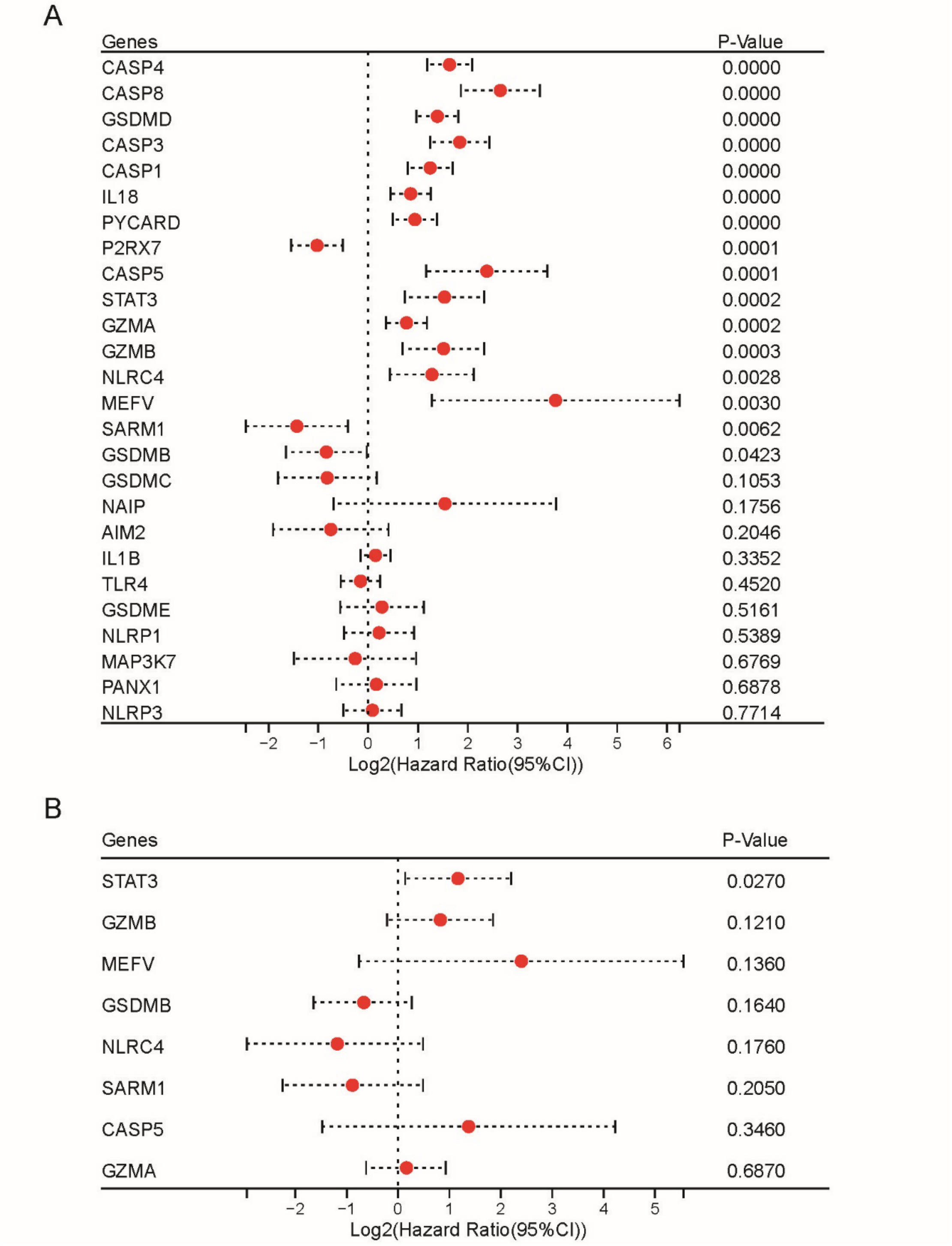

Supplementary Figure 2. Univariate (A) and multivariate cox (B) regression were performed for pyroptosis-related genes.

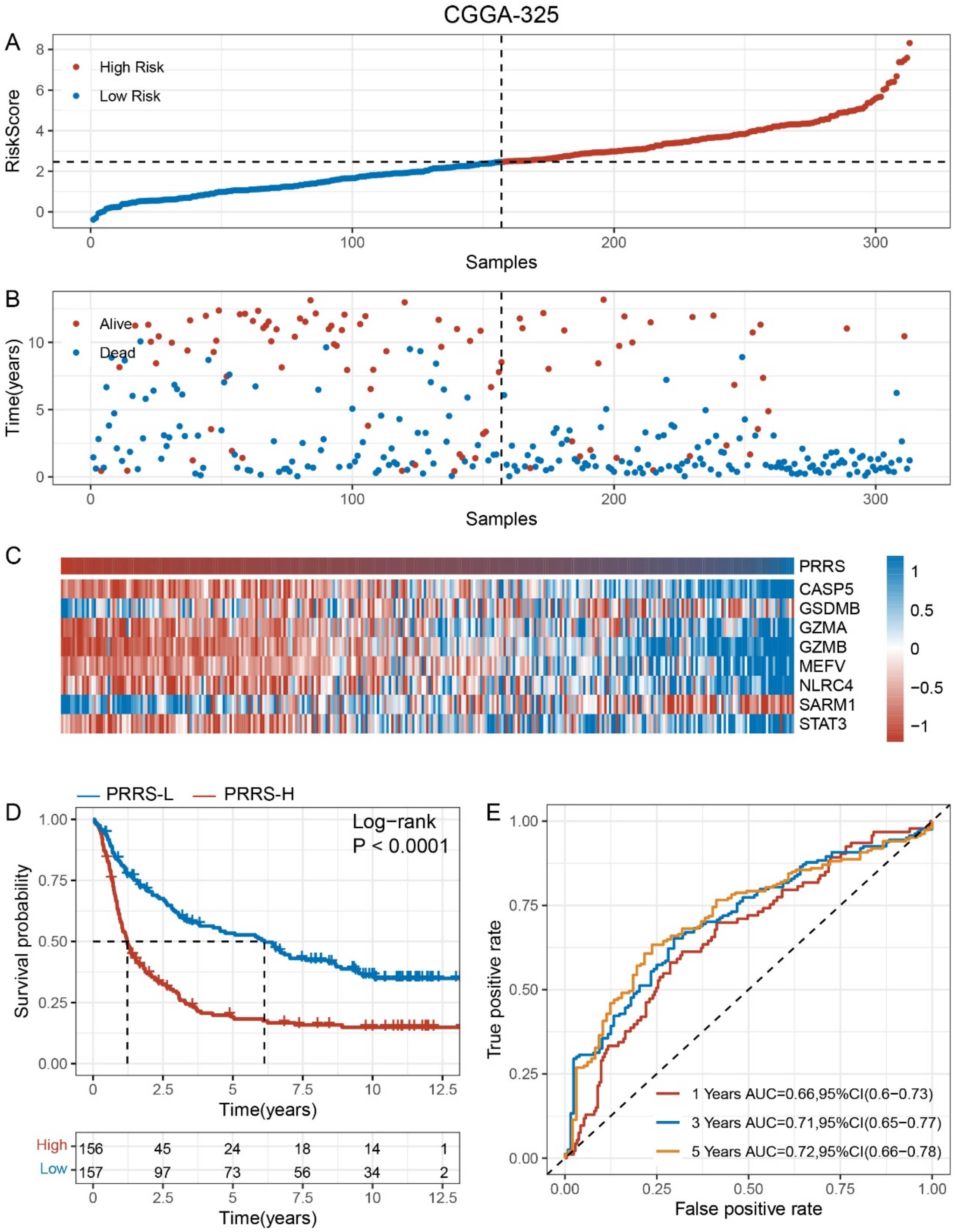

Supplementary Figure 3. Validation of PRRS in CGGA-325 cohort. (A) PRRS value in the CGGA-325 cohort. (B) Survival status in the CGGA-325 cohort. (C) Expression pattern of the PRRS genes in the CGGA-325 cohort. (D) Survival analysis of different risk group for overall survival in the CGGA-325 cohort. (E) ROC curve analysis for 1-year, 3-year and 5-year overall survival in the CGGA-325 cohort.

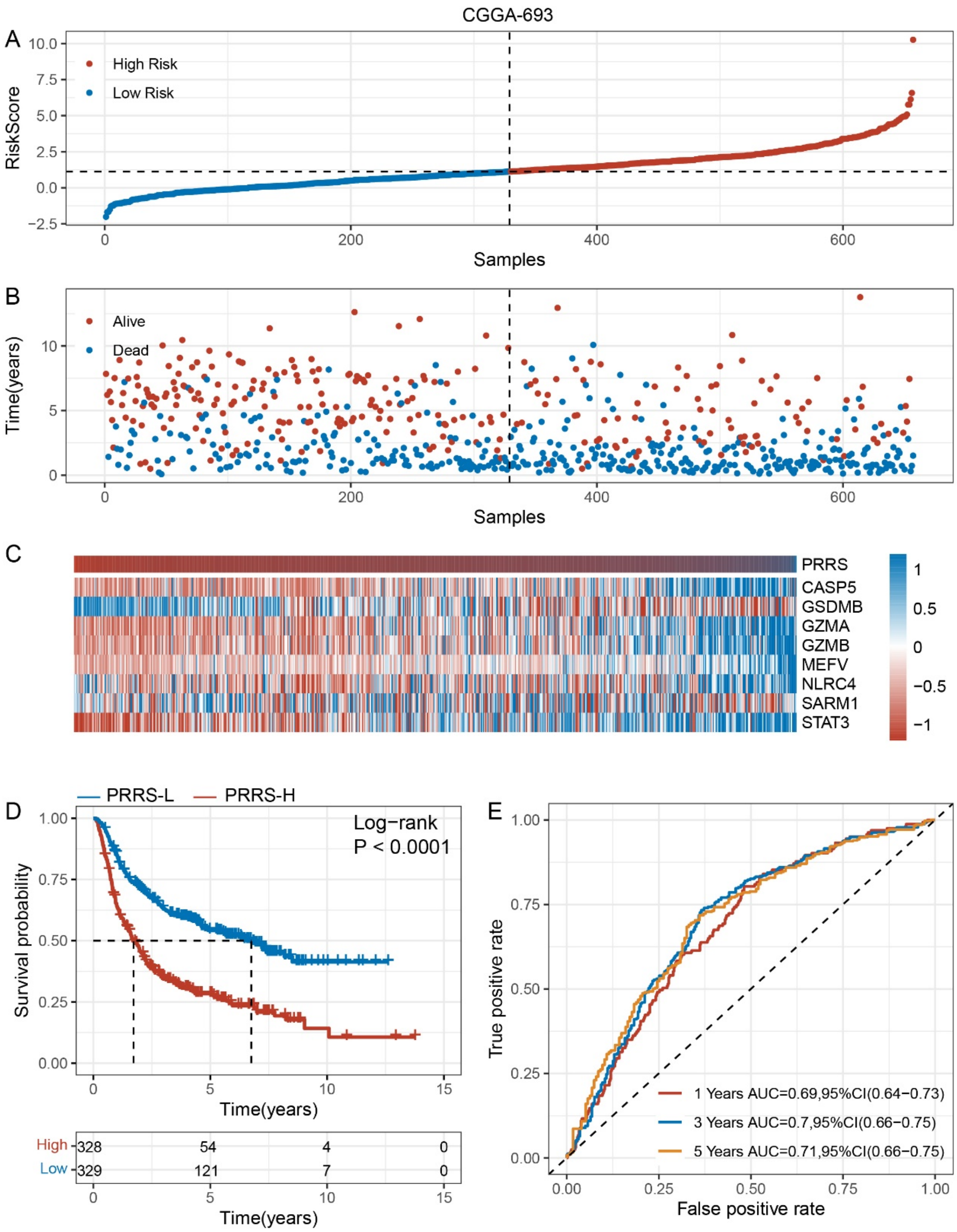

Supplementary Figure 4. Validation of PRRS in CGGA-693 cohort. (A) PRRS value in the CGGA-693 cohort. (B) Survival status in the CGGA-693 cohort. (C) Expression pattern of the PRRS genes in the CGGA-693 cohort. (D) Survival analysis of different risk group for overall survival in the CGGA-693 cohort. (E) ROC curve analysis for 1-year, 3-year and 5-year overall survival in the CGGA-693 cohort.

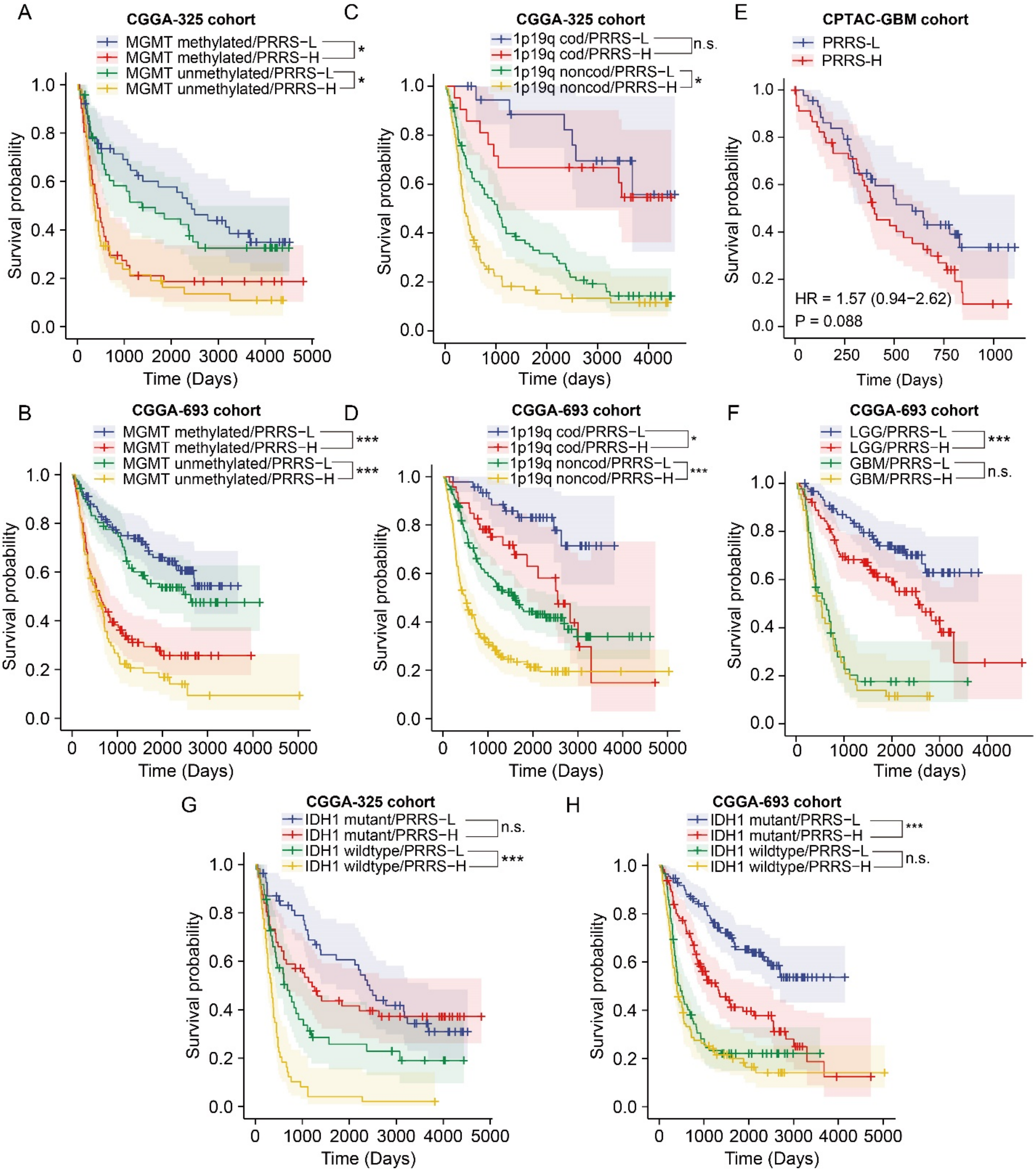

Supplementary Figure 5. Subgroup analysis in external validation cohorts. (A-B) Survival analysis of glioma patients with low and high PRRS in co-occurrence with MGMT promoter methylation or not in CGGA-325 and CGGA-693 cohorts. (C-D) Survival analysis of glioma patients with low and high PRRS in co-occurrence with 1p/19q codeletion or not in CGGA-325 and CGGA-693 cohorts. (E) Survival analysis of glioma patients with low and high PRRS in CPTAC-GBM cohort. (F) Survival analysis of glioma patients with low and high PRRS in LGG and GBM group, respectively, in CGGA-693 cohort.

(G-H) Survival analysis of glioma patients with low and high PRRS in co-occurrence with IDH1 mutation or not in CGGA-325 and CGGA-693 cohorts.

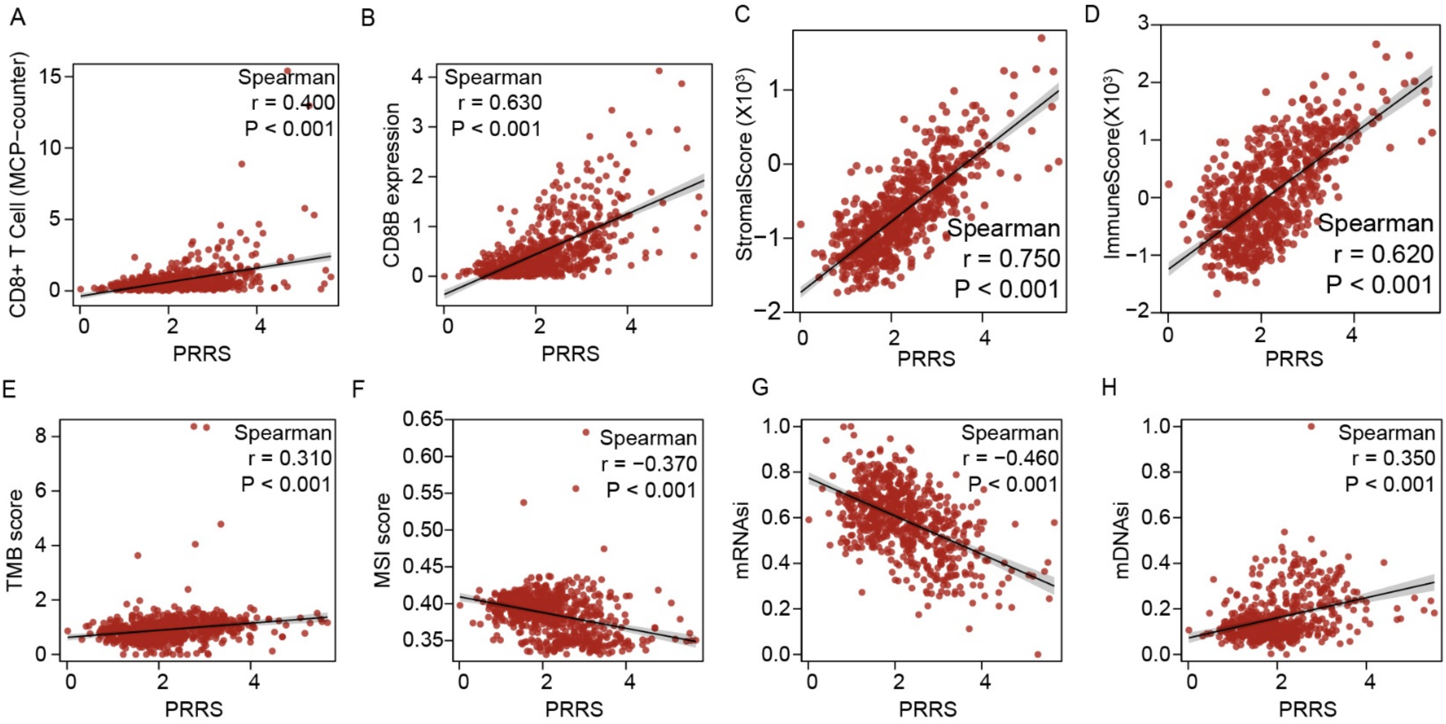

Supplementary Figure 6. The correlation between PRRS and molecular characteristics. (A) Dot graph showed the correlation between PRRS and CD8+ T cell with MCP-counter algorithm. (B) Dot graph showed the correlation between PRRS and CD8B expression. (C-H) Dot graph showed the correlation between PRRS and StromalScore, ImmuneScore, TMB score, MSI score, mRNAsi, and mDNAsi, respectively.

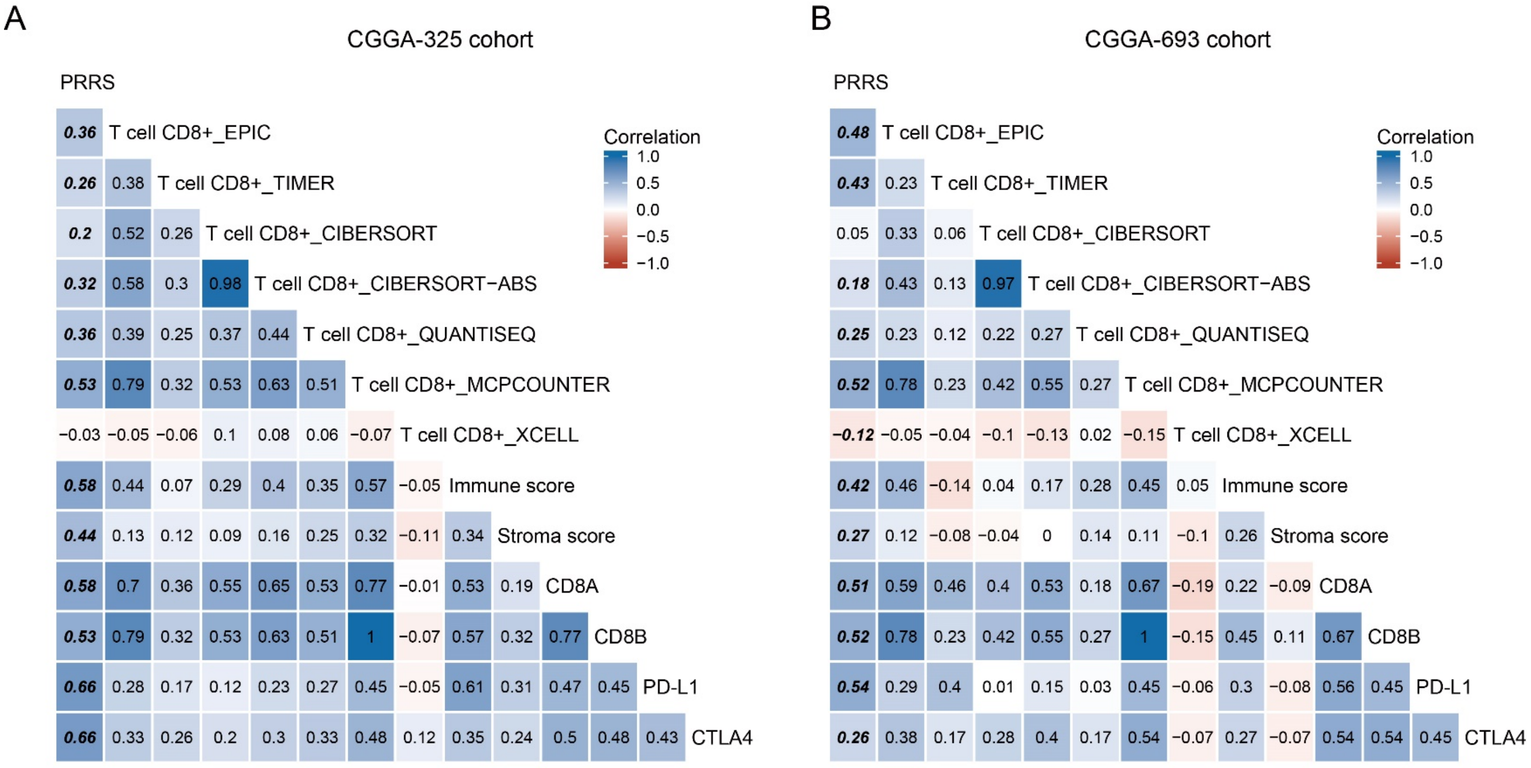

Supplementary Figure 7. The correlation between PRRS and immune characteristics in external validation cohorts. In CGGA-325 cohort (A) and CGGA-693 cohort (B), the correlation between PRRS and CD8+ T cell was analyzed using EPIC, TIMER, CIBERSORT, quanTIseq, MCP-counter, and xCell algorithms; the correlation between PRRS and immune score, stroma score, CD8A/CD8B/PD-L1/CTLA4 was analyzed.

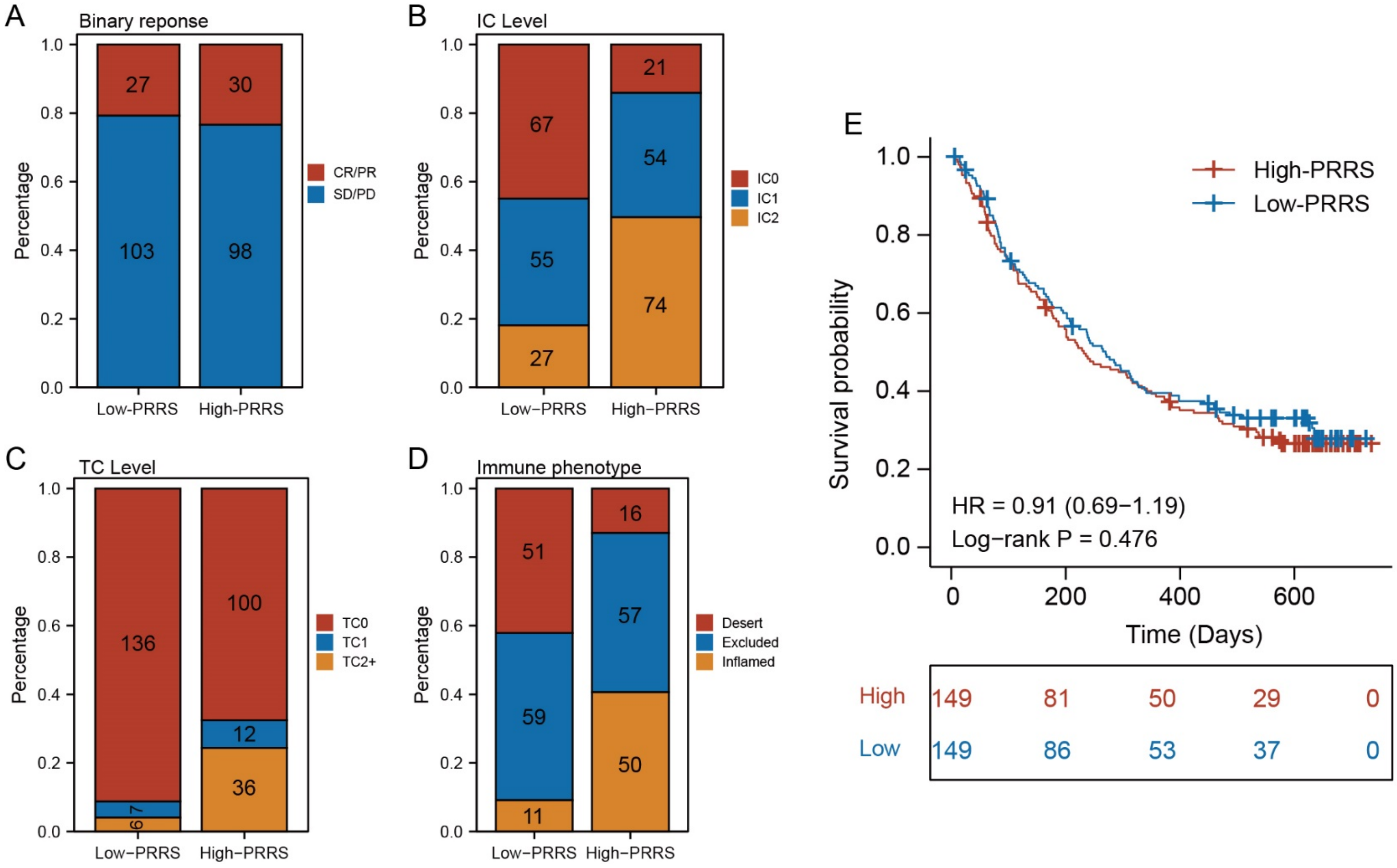

Supplementary Figure 8. Validation of PRRS value to predict immunotherapy in IMvigor210. (A) Analysis of binary response in low- and high-PRRS group. (B) Analysis of immune cell (IC) PD-L1 level in low- and high-PRRS group. (C) Analysis of tumor cell (TC) PD-L1 level in low- and high-PRRS group. (D) Analysis of immune phenotype in low- and high-PRRS group. (E) Survival analysis for overall survival between low- and high-risk group in IMvigor210.

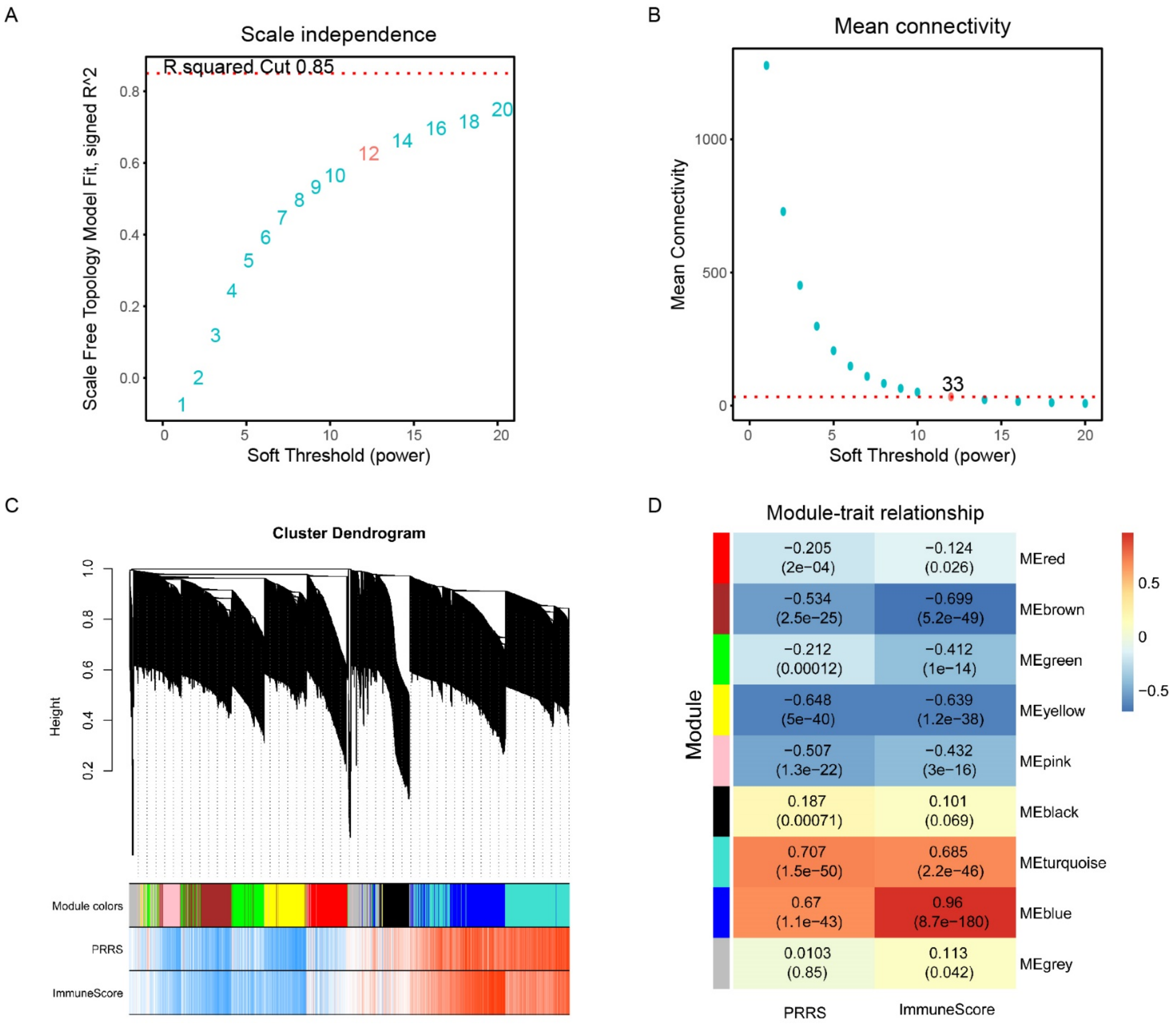

Supplementary Figure 9. Identification of canonical module genes related with PRRS by weighted gene co-expression network analysis (WGCNA) in the CGGA-325 cohort. (A-B) Determination of the most appropriate soft-thresholding power and corresponding number of mean connectivity in WGCNA in the CGGA-325 cohort. (C) Cluster dendrogram and module assignment in WGCNA in the CGGA-325 cohort. (D) Association between module and PRRS and ImmuneScore through module–trait relationship analysis in the CGGA-325 cohort.
